# Plant-derived secondary metabolites and nanotechnology: innovative strategies and emerging challenges in myocardial ischemia-reperfusion injury therapy

**DOI:** 10.3389/fphar.2025.1529478

**Published:** 2025-05-29

**Authors:** Wei Shi, Yang Xu, Jian Wei, Xiaoyu Zhang, Shuaijie Zhu, Heng Guo, Qihui Huang, Chuyao Qi, Tianfeng Hua, Yue Liu, Min Yang

**Affiliations:** ^1^ The Second Department of Critical Care Medicine, The Second Affiliated Hospital of Anhui Medical University, Hefei, Anhui, China; ^2^ Laboratory of Cardiopulmonary Resuscitation and Critical Care, The Second Affiliated Hospital of Anhui Medical University, Hefei, Anhui, China; ^3^ National Clinical Research Center for TCM Cardiology, Xiyuan Hospital of China Academy of Chinese Medical Sciences, Beijing, China; ^4^ Key Laboratory of Disease and Syndrome Integration Prevention and Treatment of Vascular Aging, Xiyuan Hospital of China Academy of Chinese Medical Sciences, Beijing, China

**Keywords:** nanoparticles, myocardial ischemia-reperfusion injury, plant-derived secondary metabolites, nanocarriers, targeting strategies

## Abstract

Therapy for acute myocardial infarction often causes myocardial ischemia-reperfusion injury (MIRI), which is characterized by oxidative stress, inflammation, and apoptosis. Traditional therapies have shown poor effectiveness because of their low absorption and inappropriate targeting. Recently, nanotechnology has emerged as a promising treatment option for MIRI. Nanocarriers, such as liposomes, polymers, inorganic nanoparticles, and hybrid nanoparticles, make therapies more effective by making drugs more stable, improving targeting accuracy and lowering side effects. Plant-derived secondary metabolites and nanoparticles, specifically those containing *Panax notoginseng* saponins and flavonoids, have been shown to work together as a therapeutic approach. These nanoparticles have antioxidant, anti-inflammatory, and anti-apoptotic properties that significantly reduce myocardial injury after reperfusion. Targeting specificity and safety limit clinical translation, even with significant technological developments in these areas. Herein, we review current studies on nanocarriers and plant-derived secondary metabolite nanoparticles for MIRI treatment, as well as potential future clinical applications and limitations.

## 1 Introduction

The incidence of ischemic heart disease, a leading cause of death and disability worldwide, has been rapidly increasing as people age and adopt unhealthy lifestyles. Public health services have been substantially affected by this trend (Safiri et al., 2022). Major symptoms of ischemic heart disease, acute myocardial infarction, are often treated with reperfusion treatments, such as percutaneous coronary intervention and coronary artery bypass grafting, which attempt to restore blood flow to the ischemic myocardium. Although reperfusion significantly reduces myocardial necrosis, it may also aggravate myocardial ischemia-reperfusion injury (MIRI), thereby compromising the patient’s prognosis (Schäfer et al., 2022). Often leading to arrhythmia and heart failure, MIRI is characterized by a range of pathogenic mechanisms, including oxidative stress, inflammation, and apoptosis (Welt et al., 2024).

Current clinical intervention strategies for MIRI face multidimensional technical challenges (Liu et al., 2023). Existing therapeutic approaches primarily include pharmacological treatments, ischemic conditioning, and physical interventions; however, their clinical efficacy remains significantly constrained. In the pharmacological domain, conventional agents such as antioxidants and calcium channel blockers suffer from limitations in targeted delivery efficiency and suboptimal pharmacokinetics, characterized by rapid systemic clearance, insufficient myocardial tissue accumulation, and adverse effects (e.g., immunosuppression associated with cyclosporine A) (Upadhaya et al., 2017). For non-pharmacological strategies, ischemic conditioning demonstrates procedural simplicity but exhibits substantial heterogeneity in clinical outcomes across randomized controlled trials (Pei et al., 2014; Donato et al., 2017) Hypothermia therapy, as a physical intervention, faces critical technical challenges in translational medicine, particularly in precise temperature control and rewarming management (Voronkov et al., 2024). Notably, although certain botanical drugs show therapeutic potential, their clinical reproducibility is hindered by compositional complexity, undefined molecular targets, and non-standardized preparation protocols, leading to inconsistent pharmacokinetic profiles (Xu et al., 2021).

Recent advances in nanodelivery systems based on plant-derived secondary metabolites (PDSMs) offer innovative avenues to overcome MIRI treatment limitations. Through rational structural design and functional modification of nanocarriers, these systems effectively address the drawbacks of conventional drug delivery, significantly enhancing targeting efficiency and biostability (Wei et al., 2022). Research priorities focus on bioactive secondary metabolites with well-characterized pharmacological properties, including baicalein (flavonoid from *Scutellaria baicalensis* Georgi), notoginsenoside R1 (triterpenoid saponin from *Panax notoginseng* (Burk.) F.H. Chen), and curcumin (polyphenol from *Curcuma longa* L.). While these compounds exhibit anti-inflammatory and antioxidant activities, their inherent low solubility and nonspecific biodistribution limit therapeutic efficacy. Current studies demonstrate that polydopamine-modified nanocarriers encapsulating baicalein activate the Nrf2-ARE pathway, markedly suppressing reactive oxygen species (ROS) accumulation in myocardial tissues (Chen et al., 2024). Mesoporous silica nanoparticles conjugated with CD11b antibody (MSN-NGR1-CD11b) enhance cardiac repair by suppressing reactive oxygen species (ROS) accumulation through activation of AKT/MAPK signaling pathways in myocardial infarction (Li H. et al., 2022). Similarly, curcumin nanoparticles significantly reduce oxidative stress markers and elevate antioxidant capacity in diabetic rats with acute myocardial injury, further supporting the efficacy of nanotechnological approaches in mitigating ROS-mediated myocardial damage (Boarescu et al., 2019b).

This work reviews recent advancements in the treatment of MIRI using PDSM nanoparticles. By focusing on their potential to enhance therapeutic efficacy, improve targeting precision, and reduce side effects, we evaluate current research and explore the clinical feasibility of PDSM nanoparticles. Additionally, we propose novel approaches for cardiovascular disease treatment.

## 2 Methods

A systematic literature search was conducted across multiple electronic databases (PubMed, Web of Science, Scopus, ScienceDirect, Embase) to identify studies published up to November 2024. Inclusion criteria encompassed *in vitro* and *in vivo* investigations evaluating the therapeutic effects of botanical drugs/PDSMs combined with nanotechnology for MIRI. The search strategy employed the following Boolean operators and keywords in titles/abstracts/keywords (nano OR nanoparticle* OR nanocarrier OR “drug delivery” OR nanophytochemical* OR nanophytomedicine* OR liposome* OR polymer* OR inorganic*) AND (“traditional Chinese medicine” OR TCM OR “Chinese herbal medicine” OR plant* OR “phytochemical extract” OR “herbal drug*” OR “botanical drug*” OR “medicinal plants” OR “plant-derived secondary metabolites”) AND (“myocardial ischemia-reperfusion injury” OR MIRI OR “cardiac ischemia-reperfusion injury” OR “acute myocardial infarction”)*. Non-English publications and studies lacking experimental validation were excluded to ensure methodological rigor.

## 3 Targeting strategies of nanoparticles

Nanotechnology demonstrates enhanced drug delivery efficiency and therapeutic efficacy in MIRI through multimodal targeting approaches. Passive targeting capitalizes on the enhanced permeability and retention (EPR) effect to promote nanoparticle accumulation in ischemic myocardium, thereby reducing systemic adverse effects (Yao et al., 2015; Li et al., 2020; Lan et al., 2022). Active targeting strategies utilize antibody or peptide-modified nanoparticles to improve cardiomyocyte-specific binding affinity and drug internalization efficiency (Wei et al., 2024). Magnetic guidance systems enable precise spatiotemporal delivery through external magnetic field manipulation (Wei et al., 2024). Stimuli-responsive platforms integrated with biomimetic designs achieve pathology-triggered drug release mechanisms, such as oxidative stress-responsive payload deployment, while maintaining enhanced biocompatibility and targeting specificity (Bae et al., 2016; Wang J. et al., 2023; Wang et al., 2023 Y.; Zhou et al., 2023). Emerging hybrid systems combining passive-active targeting synergies with multidrug co-delivery capabilities present a transformative paradigm for optimizing MIRI intervention (Sun et al., 2012; Yang et al., 2023).

## 4 Application of nanocarriers in MIRI

**FIGURE 1:**
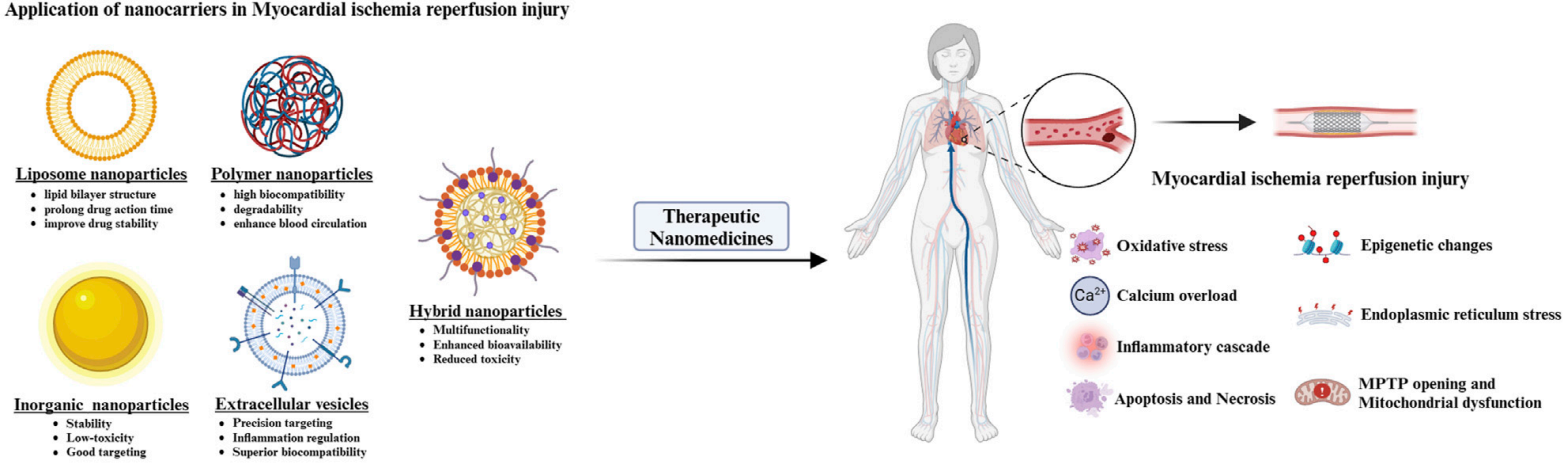
Application of nanocarriers in MIRI.

Nanotechnology offers innovative therapeutic strategies for MIRI by enabling precision drug delivery, enhancing pharmaceutical stability, and improving bioavailability. The integration of PDSMs’ inherent bioactivities with functionalized nanocarrier designs synergistically modulates oxidative stress, inflammatory responses, and apoptotic pathways, thereby overcoming the limitations of conventional treatment modalities. The following sections review several common nanocarriers applied in MIRI intervention, detailing their structural configurations and therapeutic mechanisms (Table 1; Figure 1).

**TABLE 1:** Summary of the application and advantages of different types of nanoparticles in MIRI.

Type of nanocarriers	Plant-derived secondary metabolites	Models	Efficacy	Safety	References
Neutrophil membrane-camouflaged MSN	Allicin	*In vitro*: Rat cardiac microvascular endothelial cells (CMECs) hypoxia-reoxygenation (H/R) injury model *In vivo*: Wistar rat MIRI model	Improved cardiac function, reduced myocardial infarct size, inhibition of ferroptosis, enhanced microvascular function, antioxidant and anti-inflammatory effects	Hemolysis rate <5% (at 100 μg/mL); no pathological damage in major organs; no significant toxicity to CMECs	Li et al. (2024)
Neutrophil decoys	18β-Glycyrrhetinic acid	Mouse MIRI model	Suppressed oxidative stress, reduced myocardial infarct size, improved cardiac function, blocked HMGB1-mediated inflammatory cascade	No hemolysis or coagulation abnormalities; no pathological damage in major organs; no significant toxicity to cardiomyocytes or ECs	Han et al. (2024)
PLGA-PEG nanoparticles	Orientin, Vitexin, Quercetin	*In vitro*: H9c2 cardiomyocyte H/R injury model *In vivo*: SD rat isoproterenol (ISO)-induced acute myocardial ischemia model	Improved cardiac function, reduced myocardial injury, regulated oxidative stress, anti-inflammatory effects	Hemolysis rate <5% (at 100 μg/mL); no pathological damage in major organs; no significant toxicity to H9c2 cells	Jing et al. (2024)
PLGA nanoparticles	Dihydromyricetin	*In vitro*: H_2_O_2_-induced oxidative stress model in rat H9c2 cardiomyocytes *In vivo*: SD rat pharmacokinetic model	Increased cardiomyocyte survival, reduced oxidative damage, improved bioavailability, optimized pharmacokinetic parameters	No significant cytotoxicity; excellent biocompatibility of PLGA material	Du et al. (2024)
Carbon dots	Curcumae Radix	*In vitro*: H_2_O_2_-induced oxidative stress model in H9c2 cardiomyocytes *In vivo*: SD rat ISO-induced acute myocardial infarction (AMI) model	Improved cardiac function, reduced myocardial infarct size, restored ATPase activity, significantly lowered blood lipid levels	Low cytotoxicity (H9c2 cell viability >95% at ≤125 μg/mL); no reported cardiac pathological damage	Dong et al. (2024)
Salvia-derived exosome-like nanoparticles	Salvia miltiorrhiza	C57BL/6 mouse MIRI model	Promoted endothelial cell proliferation/migration, enhanced myocardial angiogenesis, improved cardiac function, reduced myocardial fibrosis and inflammation	No histological signs of inflammation or injury in organs; excellent systemic compatibility after intravenous injection	Zhang et al. (2024)
Magnesium Ion-Doped Mesoporous Bioactive Glasses	Gallic Acid	C57BL/6 mouse MIRI model	ROS scavenging, reduced cardiomyocyte apoptosis, promoted angiogenesis, suppressed inflammation, improved cardiac function	No significant toxicity to HL-1 cardiomyocytes or HUVECs at ≤100 μg/mL; good hemocompatibility; no hemolysis or organ damage	Yu et al. (2024)
PEG-modified cationic liposomes	Apigenin	*In vitro*: ISO-treated H9C2 cells simulating myocardial ischemic injury *In vivo*: SD rat ISO-induced myocardial ischemic injury model	Reduced cardiomyocyte oxidative stress, decreased inflammatory factor secretion, inhibited apoptosis, reduced infarct size, improved ECG abnormalities	No significant cytotoxicity; excellent blood compatibility; no organ toxicity	Liu et al. (2024)
PEG-PPS self-assembled nanomicelles	Tilianin	H9c2 cardiomyocyte H/R injury model	Reduced inflammatory factors and oxidative stress, inhibited apoptosis	No significant toxicity to H9c2 cardiomyocytes at ≤5 μg/mL	Wang et al. (2018)

Synthetic nanocarriers enable efficient drug loading and controlled release through material engineering design, primarily including polymer-based nanocarriers (e.g., poly (lactic-co-glycolic acid), PLGA), inorganic nanocarriers, and lipid-based systems. Du et al. engineered PLGA nanoparticles incorporating dihydromyricetin (DMY, a flavonoid metabolite derived from *Ampelopsis grossedentata* (Hand.-Mazz.) W.T. Wang), which demonstrated enhanced oral bioavailability. These DMY-PLGA nanoparticles attenuated oxidative damage by activating the PGC1α/PPARα pathway (Du et al., 2024). Jing et al. formulated nanoparticles (OVQ-NPs) containing bioactive flavonoids (hyperoside, vitexin, quercetin) from *Polygonum orientale* L., which regulated oxidative stress and inflammatory pathways through sustained-release properties, effectively reducing myocardial enzyme levels and suppressing apoptosis (Jing et al., 2024). Yu et al. designed magnesium-doped mesoporous bioactive glass nanoparticles (MgNPs/GA) loaded with gallic acid (GA, a polyphenolic metabolite from *Rhus chinensis* Mill.), achieving dual-phase myocardial protection through early-phase ROS scavenging and late-phase Mg^2+^-mediated angiogenesis promotion with macrophage M2 polarization (Yu et al., 2024). Carbon dots derived from carbonized *Curcuma longa L.* rhizomes (CRC-CDs) directly enhanced myocardial antioxidant capacity and inhibited apoptosis, demonstrating the innovative potential of nanoscale botanical drug formulations (Dong et al., 2024). Additionally, PEGylated liposomes (P-CLP-A/R) loaded with apigenin (a flavonoid from *Apium graveolens* L.) synergistically modulated the RAGE/NF-κB pathway through targeted delivery, significantly ameliorating ischemic myocardial injury (Liu et al., 2024).

Extracellular vesicles represent biomimetic nanocarriers that enhance drug targeting and biocompatibility by mimicking biological components. Zhang et al. isolated exosome-like nanoparticles from *Salvia miltiorrhiza* Bunge, which promoted endothelial cell migration and myocardial neovascularization through inherent pro-angiogenic activity, offering a non-invasive therapeutic strategy for reperfusion injury (Zhang et al., 2024). These exosomes inherit bioactivity from parent cells while exhibiting cost-effectiveness and high yield, highlighting clinical translation potential. For composite nanocarriers, Li et al. engineered neutrophil membrane-camouflaged mesoporous silica nanoparticles (AL@MSNs@NM) that leveraged natural neutrophil interactions with inflamed myocardial microvascular endothelial cells. This system enabled precision delivery of allicin (a sulfur-containing compound from *Allium sativum* L.), inhibiting ferroptosis and upregulating PECAM-1 expression to improve cardiac function and reduce infarct size (Li et al., 2024). Similarly, Han et al. developed neutrophil degranulosomes (NDs) loaded with 18β-glycyrrhetinic acid (GA, a triterpenoid saponin from *Glycyrrhiza uralensis* Fisch.), which achieved synchronized mitigation of oxidative stress and inflammation via H_2_O_2_-responsive release, effectively attenuating myocardial fibrosis and remodeling (Han et al., 2024). These hybrid systems overcome limitations of single-component carriers by integrating biological membrane functionality with synthetic material-controlled release properties.

## 5 Plant-derived secondary metabolite nanoparticles in MIRI

The increasing recognition of PDSMs in the treatment of MIRI has highlighted the significant therapeutic potential of botanical drug extracts, which possess strong anti-inflammatory, antioxidant, anti-apoptotic, and cardiovascular-protective properties (Dong et al., 2023). Thus, PDSMs are promising therapeutic approaches for the treatment of MIRI. Concurrently, the rapid advancement of nanotechnology has opened new avenues for research and clinical applications of PDSMs, particularly in two key areas.

Processing drugs into nanoscale suspensions or co-crystals markedly increases their surface areas. This approach enhances the solubility of these drugs and improves their chemical stability. For example, studies have demonstrated that converting curcumin and quercetin into nanodispersions significantly boosts their bioavailability and pharmacological efficacy. This nanoprocessing technology effectively addresses the inadequate absorption of PDSMs by the body (Gao et al., 2010; Li H. et al., 2021).

Nanoparticles are often used for the targeted delivery of PDSMs. Unlike synthetic drugs, PDSMs often non-selectively affect multiple organ systems. However, these natural chemicals present significant therapeutic challenges, including low absorption rates, poor chemical stability, limited permeability, and risk of liver and kidney damage. Nanotechnology has distinct benefits in addressing these difficulties, as nanoparticle-based delivery methods may significantly enhance the bioavailability and therapeutic efficiency of PDSMs while reducing side effects. This precise delivery approach addresses long-standing issues in PDSMs treatment and offers new possibilities for the therapeutic use of potent botanical drug medications (Xie et al., 2024).

Nanotechnology has provided new prospects for expanding the study of PDSMs while offering technical support for their clinical application. The integration of several PDSMs with nanotechnology has been successfully implemented in therapeutic investigations targeting MIRI, with outstanding results in the targeted distribution of critical components. These include ginsenoside, puerarin, tanshinone IIA, baicalin, triptolide, and ligustrazine (Kim et al., 2018; Yan et al., 2019; Xu et al., 2020; Mi et al., 2021; Yalikong et al., 2021; Zhu et al., 2021). Nanoparticle delivery technologies significantly increase the targeting precision and therapeutic effectiveness of these drugs while effectively mitigating adverse effects.

**FIGURE 2:**
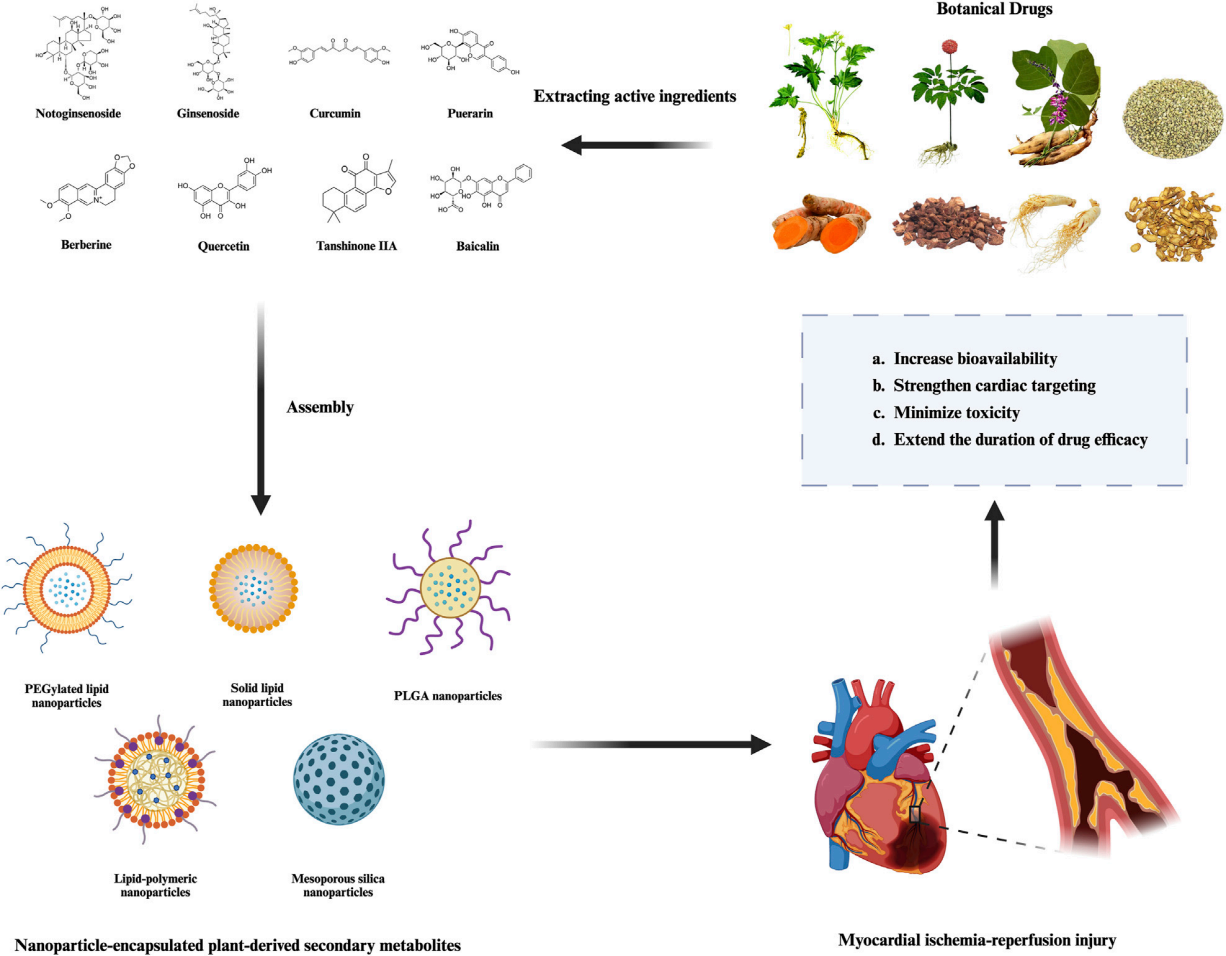
Therapeutic advantages of nanoparticle-encapsulated plant-derived secondary metabolites in MIRI.

**FIGURE 3:**
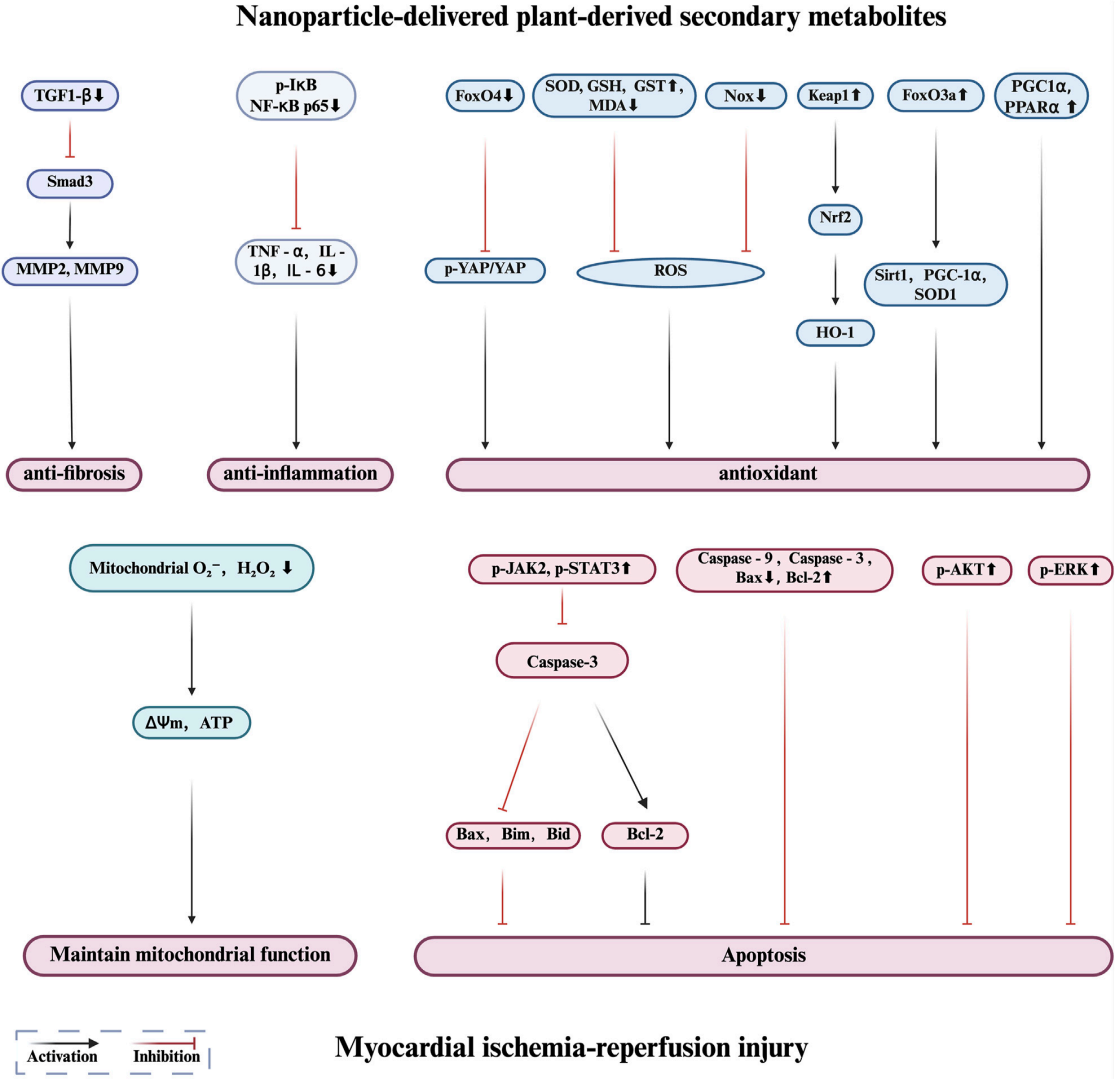
Protective mechanisms of nanoparticle-delivered plant-derived secondary metabolites in myocardial ischemia reperfusion injury.

The following section introduces several representative PDSMs with nanocarriers that are currently under investigation, along with examples of their applications and mechanisms in the treatment of MIRI (Table 2; Figures 2, 3). The successful implementation of these nanoparticles not only validates the potential of nanotechnology in enhancing the efficacy of botanical drugs but also provides valuable insights into the modernization of botanical drugs and its integration with precision medicine.

**TABLE 2:** Mechanisms and therapeutic advantages of nanocarriers delivering plant-derived secondary metabolites in myocardial ischemia-reperfusion injury.

Extract type	Nanocarrier type	Model	Drug treatment	Effects	References
*Panax notoginseng* (PNS, total extract)	Core-shell hybrid liposomal vesicles (HLV)	Rat AMI model	PNS (30 mg/kg, po); PNS-HLV (30 mg/kg, po) Duration: Pre-treatment for 10 days	Reduces oxidative stress; Long-term stability (4°C, 12 months); High encapsulation efficiency; Enhanced sustained-release properties	Zhang et al. (2012)
Salvianolic acid B (Sal B, monomer) + *Panax notoginseng* (PNS, total extract)	RGD-modified lipid-polymer hybrid nanoparticles (RGD-LPNs)	Rat AMI model	Free Sal B (30 mg/kg, iv); Free PNS (30 mg/kg, iv); RGD-S/P-LPNs (Sal B 10 mg/kg, PNS 10 mg/kg, iv) Duration: Pre-treatment for 10 days, observation for 3 days post-surgery	Significantly reduces myocardial infarction area; Improves cardiac drug distribution and prolongs plasma circulation time; Sustained release *in vitro* with no significant cytotoxicity	Qiu et al. (2017)
Notoginsenoside R1 (NGR1, monomer)	Mesoporous silica nanoparticles conjugated with CD11b antibody (MSN-CD11b antibody)	*In vivo*: Mouse myocardial infarction (MI) model *In vitro*: H9c2 cells and primary cardiomyocytes under oxygen-glucose deprivation	NGR1: *In vivo* 40 mg/kg (iv, oral dose equivalent to nano-group); *In vitro* 100 μmol/L (pre-treatment for 30 min) MSN-NGR1-CD11b antibody: 267 ng/kg (iv) Duration: Single dose 24 h post-surgery, observation for 4 weeks	Improves cardiac function, reduces infarction area and collagen deposition; Inhibits cardiomyocyte apoptosis, modulates macrophage phenotypes and inflammatory factors; Promotes angiogenesis via AKT, MAPK, and Hippo signaling pathways	Li et al. (2022a)
*Panax japonicus* (C.A.Mey.) Hoo & Tseng (Araliaceae) (total extract)	Silver nanoparticles (Ag@*P.japonicus*)	Wistar rat ISO-induced MI model	Ag@*P. japonicus:* 50, 100 μg/kg Duration: Pre-treatment for 14 days, ISO administration for 2 days	Ameliorates ECG abnormalities, reduces heart/body weight ratio and infarction area; Alleviates myocardial injury and oxidative stress; Inhibits inflammatory cytokines and apoptosis; Regulates PI3K/Akt/mTOR and Keap1/Nrf2/HO-1 pathways	Xu et al. (2024)
Ginsenoside Rg3 (monomer)	ROS-responsive polymeric nanoparticles (PEG-b-PPS)	*In vivo*: SD rat MIRI model *In vitro*: H9c2 cells under H/R	*In vitro*: Rg3 (10 nM) *In vivo*: PEG-b-PPS-Rg3 (0.5 mg Rg3/100 μL, intramyocardial injection) Duration: *In vitro* 24 h; Single dose post-reperfusion, observation for 2 h	*In vitro*: Enhances H9c2 cell viability post-H/R injury, reduces ROS generation and apoptosis; *In vivo*: Reduces myocardial infarction area, improves cardiac function, alleviates myocardial injury; Attenuates oxidative stress, inflammation, and fibrosis (downregulates TGF-β/Smad); Targets FoxO3a to activate anti-apoptotic pathways	Li et al. (2020)
Curcumae Radix Carbonisata (CRC, total extract)	Carbon dots (CDs)	*In vivo*: SD rat ISO-induced MI model *In vitro*: H9c2 cells under H_2_O_2_ injury	*In vivo*: CRC-CDs (1.75–7 mg/kg/day, po) *In vitro*: CRC-CDs (31.25–125 μg/mL) Duration: *In vivo* pre-treatment for 14 days + ISO induction for 2 days; *In vitro* pre-treatment for 24 h	Ameliorates ECG abnormalities, reduces myocardial infarction area; Alleviates myocardial injury and oxidative stress; Inhibits cardiomyocyte apoptosis; Improves mitochondrial ATPase activity	Dong et al. (2024)
Curcumin (monomer)	Polymeric nanoparticles	Wistar rat ISO-induced MI model	Curcumin (100–200 mg/kg, po); Curcumin nanoparticles (100–200 mg/kg, po) Duration: Pre-treatment for 15 days, ISO induction for 2 days	Ameliorates ECG abnormalities; Reduces myocardial injury and oxidative stress; Alleviates cardiomyocyte edema, inflammatory infiltration, and fibrosis	Boarescu et al. (2019b)
Curcumin (monomer)	Polymeric nanoparticles	Streptozotocin (STZ)-induced diabetic + ISO-induced MI rat model	Curcumin (200 mg/kg, po); Curcumin nanoparticles (200 mg/kg, po) Duration: Pre-treatment for 7 days (pre-DM induction) + 15 days (post-DM induction), ISO induction for 2 days	Reduces myocardial injury; Lowers inflammatory cytokines; Improves oxidative stress	Boarescu et al. (2019a)
Curcumin (monomer)	Polylactic acid nanoparticles (CurNisNp)	Guinea pig ISO-induced MI model	CurNisNp (10, 21 mg/kg, po) Duration: Pre-treatment for 7 days, ISO induction for 2 days	Ameliorates ECG abnormalities; Reduces myocardial injury and oxidative stress; Alleviates cardiomyocyte necrosis and inflammatory infiltration	Nabofa et al. (2018)
Puerarin (PUE, monomer)	Ischemic myocardium-targeting peptide (IMTP) and triphenylphosphonium cation (TPP)-co-modified liposomes (PUE@T/I-L)	*In vitro*: H9c2 cells under H/R *In vivo*: C57BL/6 mouse MIRI model	*In vitro*: PUE@T/I-L (20 μM, administered during reoxygenation for 12 h) *In vivo*: PUE@T/I-L (15 mg/kg, iv, 5 min pre-reperfusion) Duration: *In vitro* 12 h; Single dose *In vivo*	*In vitro*: Inhibits mitochondrial permeability transition pore (mPTP) opening, reduces ROS generation, enhances SOD activity, improves cell viability post-H/R injury; *In vivo*: Reduces myocardial infarction area, alleviates myocardial injury; Improves mitochondrial morphology, inhibits apoptosis, targets ischemic myocardium mitochondria	Wang et al. (2024a)
Puerarin (PUE, monomer)	RGD-modified and PEGylated solid lipid nanoparticles (RGD/PEG-PUE-SLN)	SD rat AMI model (left anterior descending coronary artery ligation)	Free PUE (50 mg/kg, iv); RGD/PEG-PUE-SLN (50 mg/kg, iv) Duration: Single dose, observation for 36 h	Prolongs systemic circulation time; Enhances cardiac drug concentration, reduces myocardial infarction area; No significant cytotoxicity *In vitro*; Improves myocardial histopathology *In vivo*	Dong et al. (2017)
Quercetin (monomer)	Mesoporous silica nanoparticles (Q-MSNs)	*In vivo*: SD rat MIRI model *In vitro*: Neonatal SD rat primary cardiomyocytes under H/R	*In vitro*: Free Quercetin (10–40 μM); Q-MSNs (10–40 μM) *In vivo*: Free Quercetin (40 μmol/L, po); Q-MSNs (40 μmol/L, po) Duration: *In vivo* pre-treatment for 10 days; *In vitro* pre-treatment for 24 h	Activates JAK2/STAT3 pathway, inhibits cardiomyocyte apoptosis; Reduces myocardial infarction area, improves ventricular remodeling; Ameliorates oxidative stress; *In vitro*: Enhances cell viability post-H/R injury	Liu et al. (2021a)
Quercetin (monomer)	Poly (lactic-co-glycolic) acid nanoparticles (PLGA-NPs)	*In vitro*: H9c2 cardiomyocytes under H/R	Free Quercetin (1–10 μM); PLGA-Quercetin NPs (1–10 μM) Duration: 24 h pre-treatment	Inhibits mitochondrial superoxide generation; Enhances cell viability, reduces thiol oxidative damage; Preserves mitochondrial membrane potential and ATP synthesis, maintains mitochondrial respiration	Lozano et al. (2019)
Berberine (BBR, monomer)	Platelet membrane-coated PLGA nanoparticles (BBR@PLGA@PLT NPs)	SD rat MI model	BBR@PLGA@PLT NPs (0.8 mg BBR/kg, iv; additional doses on days 0, 3, 6 post-MI) Duration: Observation for 3 days (inflammatory markers) to 28 days (cardiac function)	Reduces M1 macrophages (CD86^+^) and increases M2 macrophages (CD206+) in infarct zone; Inhibits inflammatory cytokine secretion; Improves cardiac function and scar elasticity at 28 days; Targets infarcted myocardium with minimal liver uptake and organ toxicity	Zhu et al. (2023a)
Berberine (BBR, monomer)	PEGylated liposomes (BB-lip)	*In vivo*: C57BL/6 J mouse MI model *In vitro*: RAW 264.7 macrophages under LPS stimulation	Free BBR (1.5 mg/kg, iv; additional doses on days 0, 3, 6 post-MI) BB-lip (1.5 mg/kg, iv; additional doses on days 0, 3, 6 post-MI) Duration: *In vitro* 2 h pre-treatment +12 h LPS stimulation; *In vivo* observation for 28 days	*In vitro*: Free BBR inhibits IL-6 secretion, BB-lip shows no inhibition due to sustained release; *In vivo*: Preserves LVEF at 28 days, reduces left ventricular end-diastolic/systolic volumes, inhibits fibrosis and inflammatory infiltration; Liposomes target infarct zone, co-localize with macrophages, reduce systemic toxicity	Allijn et al. (2017)
Tanshinone IIA (monomer)	Methoxy polyethylene glycol-polylactic acid-vitamin E succinate nanoparticles (mPEG-PLA-TPGS NPs)	C57BL/6 mouse MI model	Free Tanshinone IIA (1, 10 mg/kg, iv); Tanshinone IIA-NPs (0.5 mL/kg, iv) Duration: Post-surgery administration for 5 consecutive days, observation for 4 weeks	Improves cardiac function; Inhibits myocardial fibrosis; Exerts anti-inflammatory and anti-apoptotic effects via NF-κB pathway inhibition (reduced phosphorylated IκB and nuclear p65)	Mao et al. (2018)

### 5.1 Notoginsenoside

Notoginsenoside R1 (NGR1), a triterpenoid saponin primarily isolated from the dried roots and rhizomes of *Panax notoginseng* (Burk.) F.H.Chen (Araliaceae family, *Panax* genus), demonstrates potent cardioprotective effects against MIRI (Zhu and Wan, 2023). NGR1 suppresses the transforming growth factor-β-activated kinase 1-JNK/p38 signaling pathway, reducing inflammatory cytokine production, considerably reducing infarct size, and enhancing heart function (Zeng et al., 2023). NGR1 reduces apoptosis by regulating critical signaling pathways, including AKT and JAK2, thereby enhancing its cardioprotective effects (Lei et al., 2022; Xu et al., 2022).

Owing to its anti-inflammatory, antioxidant, and anti-apoptotic properties, NGR1 has been extensively investigated. MIRI-related damage is primarily caused by oxidative stress and myocardial cell death. Superoxide dismutase, which lowers ROS generation and shields cardiac cells from oxidative stress (Tong et al., 2019), is an antioxidant enzyme that NGR1 promotes. Moreover, NGR1 helps stabilize mitochondrial membrane potential, thereby lowering mortality and shielding against heart injury (Yan et al., 2021).

Although notoginsenoside has enormous therapeutic potential, its low bioavailability, poor water solubility, and sensitivity to metabolic breakdown restrict its therapeutic uses (Li et al., 2007). To overcome these limitations, researchers have devised mesoporous silica nanoparticle-based delivery systems, including NGR1. This method, together with CD11b antibodies, focuses on injured regions after myocardial infarction, thereby increasing the local concentration of NGR1 in the myocardium. This method reduces myocardial reperfusion damage, enhances heart performance, and increases the antioxidant, anti-inflammatory, and anti-apoptotic actions of NGR1. Moreover, the treatment controls energy metabolism and induces angiogenesis (Li H. et al., 2022). However, the long-term biosafety profile of Notoginsenoside R1-loaded mesoporous silica nanoparticles conjugated with CD11b antibody and their potential off-target effects on non-infarcted tissues expressing CD11b remain uncharacterized, necessitating further preclinical validation.

NGR1 was used in a core-shell hybrid liposome nanoparticle system to increase the bioavailability of notoginsenoside. After oral delivery, this method greatly enhanced the pharmacological effects of notoginsenosides, thereby lowering the severity of myocardial infarction, controlling oxidative stress, and increasing myocardial cell survival rates (Zhang et al., 2012). This study proposes a novel approach to enhance the delivery of notoginsenoside. Notably, the current findings are limited by the lack of pharmacokinetic evaluations to delineate the absorption mechanisms of panax notoginsenoside-encapsulated core-shell hybrid liposomal delivery systems and insufficient toxicological monitoring over extended durations, which necessitates further investigation prior to human trials.

Researchers incorporated notoginsenoside and salvianolic acid B into an RGD-modified dual-drug delivery system. This technique greatly improved myocardial targeting, lowered infarction size, and improved heart function (Qiu et al., 2017). With significant consequences for future cardiovascular disease treatments, this dual-drug delivery system epitomizes the benefits of multi-target therapeutic approaches. The limitations of this study include that the experimental findings were primarily derived from a rat model of acute myocardial ischemia, which may not fully replicate the pathophysiological complexity of human cardiovascular disease, potentially restricting the direct clinical translatability of the results.

### 5.2 Ginsenoside

Ginsenosides, a class of dammarane-type and oleanane-type triterpenoid saponins characteristic of *Panax* genus (Araliaceae) plants, are secondary metabolites predominantly distributed in the roots and rhizomes of *Panax ginseng* C.A.Mey., *Panax notoginseng* (Burk.) F.H.Chen, and *Panax quinquefolius* L. These bioactive compounds exhibit multi-pharmacological properties including anti-inflammatory, antioxidant, antitumor, and neuroprotective activities. Common ginsenosides, such as Rg1, Rg3, Rb1, and Rc, affect many physiological processes by regulating cellular signaling pathways (Chen, 2020). In addition to their potential in treating neurodegenerative diseases and cancer, ginsenosides have attracted attention for their protective effects on the cardiovascular system (Sarhene et al., 2021).

Qin et al. have demonstrated that ginsenoside Rb1 significantly improved MIRI by reducing myocardial autophagy through the modulation of the PI3K/Akt/mTOR signaling pathway (Qin et al., 2021). Similarly, Wang et al. have reported that ginsenoside Rd alleviates MIRI by reducing inflammation and apoptosis via inhibition of the PI3K/AKT signaling pathway (Wang et al., 2024c). Ye et al. have found that ginsenoside Re protects cardiomyocytes by inhibiting ferroptosis by regulating the miR-144–3p/SLC7A11 signaling pathway (Ye et al., 2023). Furthermore, Xue et al. have found that ginsenoside Rc mitigates MIRI by activating the sirtuin 1 (SIRT1 pathway), thereby reducing mitochondrial oxidative stress and apoptosis (Xue et al., 2023). Li et al. have demonstrated that ginsenoside Rg2 significantly improves MIRI by inhibiting necrosis by modulating the transforming growth factor-β-activated kinase one signaling pathway (Li Y. et al., 2022). Huang et al. have demonstrated that the ginsenoside Rb2 reduces MIRI by inhibiting SF3A2 acetylation and regulating Fscn1 selective splicing (Huang et al., 2023). Another study by Xue et al. has revealed that ginsenoside Rb2 improves cardiac function by reducing oxidative stress through SIRT1 activation (Xue et al., 2020). Collectively, these studies highlight the diverse cardioprotective mechanisms of ginsenosides against MIRI.

Despite their promising therapeutic effects, the clinical application of ginsenosides is limited owing to their low water solubility and poor bioavailability (Pan et al., 2018). Therefore, improving their bioavailability and pharmacological efficacy has become a major focus of current research. Recently, nanotechnology has been used to enhance the stability, targeting, and circulation time of ginsenoside-based drugs, thereby boosting their clinical efficacy. Nanoparticles not only improve the solubility of hydrophobic ginsenosides, such as Rg3, but also concentrate drug effects at specific disease sites through targeted delivery, thereby minimizing side effects. For example, Xu et al. developed Ag@*P. japonicus* nanoparticles by integrating silver nanoparticles with *Panax* japonicus extract. These nanoparticles reduced myocardial apoptosis and inflammation, enhanced antioxidant enzyme activity (e.g., superoxide dismutase and glutathione peroxidase), decreased infarct size, and improved cardiac function in an isoproterenol-induced myocardial infarction model. This study underscored the potential of nanotechnology in enhancing the efficacy and stability of ginsenosides (Xu et al., 2024). Li et al. investigated a ROS-responsive nanoparticle delivery system for ginsenoside Rg3. By targeting the FoxO3a pathway, Rg3 inhibited oxidative stress, inflammation, and fibrosis, resulting in improved cardiac function after MIRI. Nanotechnology significantly enhanced the bioavailability of Rg3, demonstrating its potential as a delivery strategy for other natural products (Li et al., 2020). Li et al. demonstrated limitations in their study by failing to comprehensively evaluate the long-term biosafety and potential immunogenicity of reactive oxygen species (ROS)-responsive nanoparticles, while the lack of validation in preclinical large animal models further constrained their clinical translatability (Li et al., 2020). Xu et al. did not address the *In vivo* pharmacokinetics or long-term bioaccumulation risks of silver nanoparticles, nor did they systematically analyze dose-dependent toxicity or stability in complex pathological microenvironments, thereby compromising a thorough assessment of therapeutic safety (Xu et al., 2024).

### 5.3 Curcumin

Curcumin, a diarylheptanoid compound (chemical formula C_21_H_20_O_6_, molecular weight 368.38 Da), is primarily isolated from the dried rhizomes of *C. longa* L (Zingiberaceae). Secondary botanical sources include rhizomes of the congeneric species *Curcuma aromatica* Salisb. And *Curcuma zedoaria* (Berg.) Rosc., albeit with lower extraction yields. In MIRI, curcumin mitigates oxidative stress by scavenging ROS and enhancing antioxidant enzyme activity. It also inhibits the production of pro-inflammatory cytokines such as TNF-α and IL-6, thereby reducing inflammation while suppressing myocardial cell apoptosis by regulating apoptosis-related proteins. Numerous studies have investigated multiple protective mechanisms of curcumin in MIRI, consistently revealing its significant cardioprotective effects.

For example, Wu et al. have demonstrated that curcumin exerts protective effects against myocardial damage by activating the PI3K/Akt/mTOR signaling pathway, providing key insights into its role in cellular signal transduction (Wu et al., 2021). Cui et al. have revealed that curcumin stimulates vascular endothelial cells to secrete fibroblast growth factor 2, thereby reducing hypoxia/reoxygenation damage to myocardial cells and reinforcing the role of curcumin in promoting myocardial repair (Cui J.-K. et al., 2024). Moreover, Cui et al. have revealed that curcumin alleviates myocardial injury by increasing endogenous H_2_S levels and regulating m6A RNA modifications, offering deeper insights into its molecular regulatory mechanisms (Cui J. et al., 2024). Zhang et al. have shown that curcumin regulates mitochondrial metabolism and inhibits apoptosis, significantly improving the outcomes of cardiopulmonary resuscitation following cardiac arrest (Zhang et al., 2022).

Nanotechnology has been widely used to enhance the bioavailability and stability of curcumin. Nabofa et al. investigated curcumin-nisin-based polylactic acid nanoparticles (CurNisNp) and found that they effectively prevented isoproterenol-induced myocardial injury by reducing oxidative stress and inflammation (Nabofa et al., 2018). Boarescu et al. used nano-curcumin particles with a PLGA carrier, which significantly improved the absorption rate of curcumin and demonstrated stronger antioxidant and anti-inflammatory effects, particularly by enhancing electrocardiogram readings and protecting cardiac function (Boarescu et al., 2019b). In another study, Boarescu et al. validated the superior performance of nano-curcumin in a diabetic MIRI model, demonstrating that it not only significantly reduced oxidative stress and inflammation but also exhibited excellent cardioprotective effects (Boarescu et al., 2019a). Additionally, Dong et al. studied carbon quantum dot nanoparticles (CRC-CDs) extracted from turmeric, which further reduced cardiac damage in MIRI by enhancing antioxidant enzyme activity and inhibiting myocardial cell apoptosis (Dong et al., 2024).

While existing studies provide valuable insights into nanomaterial-based cardioprotective strategies, several critical limitations persist. Dong et al. focused exclusively on short-term rodent models, neglecting evaluations of chronic toxicity and sustained therapeutic efficacy of carbon dots in clinically relevant myocardial ischemia paradigms, thereby limiting translational validity (Dong et al., 2024). Boarescu et al. omitted pharmacokinetic profiling of nanocurcumin, particularly its long-term bioavailability and metabolic fate, and employed a restricted experimental dose range that may inadequately characterize dose-response relationships (Boarescu et al., 2019b); their subsequent study further constrained generalizability by excluding non-diabetic myocardial infarction models and failing to address nanoparticle stability and tissue distribution dynamics (Boarescu et al., 2019a). Additionally, Nabofa et al. relied solely on guinea pig models, which exhibit marked cardiovascular physiological divergence from humans, while overlooking immunogenicity assessments and chronic safety risks of their composite nanoparticles (Nabofa et al., 2018). Collectively, these limitations highlight gaps in interspecies physiological relevance, comprehensive chronic exposure evaluations, and rigorous pharmacokinetic characterization, necessitating further research to bridge preclinical findings and clinical applicability.

### 5.4 Puerarin

Puerarin, an isoflavone C-glycoside (chemical formula C_21_H_20_O_9_, molecular weight 416.38 Da), is primarily isolated from the roots of *Pueraria lobata* (Willd.) Ohwi and *Pueraria thomsonii* Benth (Fabaceae family, *Pueraria* genus). This phytochemical demonstrates significant cardioprotective effects within the cardiovascular system, underpinning its widespread clinical application in managing hypertension and coronary artery disease (Zhou et al., 2014). Consequently, it is widely used in the treatment of cardiovascular diseases such as hypertension and coronary artery disease. Numerous studies have demonstrated the beneficial effects of puerarin on MIRI with its protective mechanisms operating at multiple levels (Zhou et al., 2021).

Puerarin regulates miR-21, reducing apoptosis and inhibiting oxidative stress, which enhances antioxidant capacity and significantly improves the survival rate of myocardial cells during MIRI (Xu et al., 2019). It further protects cardiac cells by increasing the long non-coding RNA ANRIL and blocking autophagy (Han et al., 2021). Puerarin inhibits the SIRT1/NF-κB pathway and prevents NLRP3 inflammasome activation, thereby reducing MIRI-induced inflammation (Wang et al., 2020). Puerarin also prevents ferroptosis, a unique type of cell death that reduces cardiac cell damage and provides overall cardioprotection (Ding et al., 2023).

Despite its promising therapeutic potential, its low water solubility and short half-life limit its clinical use (Wang et al., 2022). Nanoparticle carrier technologies have been developed to improve the bioavailability and targeting efficiency of PUR. For example, triphenylphosphonium cation and ischemic myocardium-targeting peptide-modified liposomes (PUE@T/I-L) were designed to target mitochondria, increasing puerarin localization in the myocardium, reducing oxidative stress, protecting myocardial cells from ischemia-reperfusion injury, and decreasing infarct size (Wang et al., 2024a). Moreover, RGD-modified and PEGylated solid lipid nanoparticles significantly prolonged the retention time of puerarin in the body, augmenting its concentration and efficacy in the heart while offering substantial protection against acute myocardial ischemia [148]. These nanoparticle carriers not only promote medication stability and controlled release but also maximize targeted distribution, highlighting the promise of puerarin in the precision treatment of cardiovascular disorders (Dong et al., 2017).

However, the following methodological limitations may constrain their translational relevance: Wang et al. did not validate the long-term biodistribution or organotoxicity of their dual-targeted liposomes in large mammals or advanced preclinical models, potentially undermining clinical translatability (Wang et al., 2024b); Dong et al. omitted systematic evaluation of RGD-modified nanoparticles’ targeting stability under dynamic pathological conditions (e.g., fibrosis or chronic inflammation), limiting generalizability of their therapeutic strategy (Dong et al., 2017).

### 5.5 Quercetin

Quercetin, a flavonol ubiquitously distributed in the plant kingdom (chemical formula C_15_H_10_O_7_, molecular weight 302.24 Da), predominantly occurs in nature as glycosidic forms such as rutin, liberating free quercetin upon acid hydrolysis or enzymatic conversion. Its principal botanical sources include the flower buds (Huaimi) of *Sophora japonica* L (Fabaceae), epidermal tissues of *Allium cepa* L (Amaryllidaceae), and fruit peel of *Malus domestica* Borkh (Rosaceae). Quercetin have demonstrated notable cardioprotective effects in MIRI therapy owing to their strong antioxidant and anti-inflammatory properties (Zhang et al., 2020). Quercetin regulates the main pathways involved in MIRI. To prevent cell death, it scavenges ROS, reduces oxidative stress, quiesces inflammatory responses, and preserves mitochondrial activity. Moreover, it influences signaling channels, including ATP-sensitive potassium channels and the nitric oxide system, which cooperate to minimize cardiac cell damage and necrosis. Many studies have demonstrated the effectiveness of quercetin in reducing cardiac damage caused by MIRI via various molecular pathways. Liu Y et al. have discovered that quercetin greatly reduced myocardial cell death and provided cardiac protection by inducing the NO system and mitochondrial ATP-sensitive potassium channels (Liu Y. et al., 2021). Chang et al. have shown that via a DNA-PKcs-SIRT5-regulated mitochondrial quality control mechanism, quercetin reduces necroptosis and, therefore, enhances heart function (Chang et al., 2024). Furthermore, Li et al. have discovered that quercetin significantly reduced oxidative stress and mitochondrial-mediated death by modulating the extracellular signal-regulated protein kinases one and 2/DRP1 signaling pathway, thereby increasing the resistance of the heart to damage. These results demonstrate the enormous therapeutic potential of quercetin in MIRI (Li F. et al., 2021).

Despite the potential therapeutic effects of quercetin, its clinical use is limited because of its physicochemical properties. Quercetin has poor water solubility and is rapidly metabolized in the body, resulting in low bioavailability and difficulty in maintaining a sufficient drug concentration and duration of action, which restricts its therapeutic effectiveness (Alizadeh and Ebrahimzadeh, 2022). To overcome these challenges, nanotechnology has been used to enhance the drug delivery performance of quercetin.

Nanotechnology has significantly improved the therapeutic efficacy of quercetin. Liu et al. have demonstrated that loading quercetin into mesoporous silica nanoparticles effectively reduced myocardial infarction size and improved cardiac physiological and biochemical functions, particularly in protecting against MIRI by activating the JAK2/STAT3 signaling pathway and inhibiting apoptosis and oxidative stress (Liu C.-J. et al., 2021). Similarly, Lozano et al. encapsulated quercetin in PLGA nanoparticles and found that this nanodelivery system exhibited stronger cardioprotective effects against oxidative stress compared with free quercetin, especially by reducing mitochondrial ROS production, maintaining mitochondrial function and preserving ATP synthesis (Lozano et al., 2019).

Although both studies provide critical evidence for the therapeutic potential of nanoparticle-mediated quercetin delivery, their methodological frameworks exhibit significant limitations. Liu et al. exclusively employed a 10-day prophylactic pretreatment regimen prior to ischemia-reperfusion (IR), failing to evaluate acute therapeutic interventions or clinically relevant post-ischemic treatment windows (e.g., intra-reperfusion or emergency post-infarction administration), thereby limiting the clinical translatability of their efficacy assessments (Liu C.-J. et al., 2021). Similarly, Lozano et al. relied solely on *in vitro* hypoxia-reoxygenation models using the H9c2 rat cardiomyoblast cell line, omitting validation in primary cardiomyocytes or pathophysiologically complex conditions (e.g., hypertension or metabolic comorbidities), which may overestimate the applicability of their findings to human cardiac pathophysiology (Lozano et al., 2019).

### 5.6 Berberine

Berberine, an isoquinoline alkaloid (chemical formula C_20_H_18_NO_4_^+^, molecular weight 336.36 Da), is predominantly isolated from the rhizomes of *Coptis chinensis* Franch (Ranunculaceae), root bark of *Berberis julianae* Schneid (Berberidaceae), and bark of *Phellodendron amurense* Rupr (Rutaceae). This phytochemical has garnered significant pharmacological attention due to its broad-spectrum bioactivities. These include anti-inflammatory, antioxidant, antibacterial, and metabolic regulatory effects (Song et al., 2020). Berberine’s potential in cardiovascular illnesses has recently received considerable attention for its preventive function against MIRI (Feng et al., 2019). Jia et al. showed that berberine greatly reduces myocardial damage by decreasing inflammation and oxidative stress, predominantly via the miR-26b-5p-mediated prostaglandin-endoperoxide synthase 2/MAPK signaling pathway (Jia et al., 2022). Long et al. have discovered that berberine improves cardioprotection by upregulating miR-340-5p and inhibiting high mobility group box 1 (HMGB1)-mediated inflammatory response (Long et al., 2023). Abdulredha et al. have demonstrated the ability of berberine to prevent MIRI by interfering with oxidative stress and inflammatory pathways (Abdulredha et al., 2021). Hu et al. have discovered that berberine decreased myocardial damage by blocking excessive autophagy via the RhoE/adenosine monophosphate-activated protein kinase pathway (Hu et al., 2024). Yang et al. have clarified this process and found that berberine suppressed cardiomyocyte ferroptosis, providing further protection (Yang et al., 2022). Overall, these findings show that berberine has a broad cardioprotective effect against MIRI by modulating inflammation, oxidative stress, autophagy, and ferroptosis.

Although berberine has significant pharmacological properties, its low water solubility and rapid metabolism restrict its clinical use and reduce its bioavailability, making it difficult to maintain therapeutic concentrations in target tissues (Wang et al., 2017). To overcome these limitations, researchers have used nanotechnology to add berberine to nanoparticle delivery methods. This approach enhances solubility, stability, and focused distribution, thereby increasing therapeutic effectiveness.

To restore heart function after myocardial infarction, Allijn et al. synthesized berberine in liposomes. Their results revealed that long-circulating liposomes enhanced the stability and bioavailability of berberine. By preventing ventricular remodeling, preserving the ejection fraction, and reducing the risk of heart failure, berberine liposomes provided significant cardioprotection after myocardial infarction. This demonstrates how liposomal technology can improve the medicinal and cardioprotective properties of berberine (Allijn et al., 2017). Zhu et al. investigated the use of platelet membrane-coated nanoparticles to treat MIRI with berberine. Their results revealed that extended drug release after reperfusion was made possible by berberine encapsulated in PLGA nanoparticles coated with a platelet membrane that was preferentially localized at the location of myocardial infarction. Platelet membrane-coated nanoparticles accumulated more in the heart tissue than standard PLGA nanoparticles, resulting in significantly fewer systemic side effects. Significant post-reperfusion cardiac function improvements resulted from this new delivery strategy, changing macrophage polarization, lowering inflammation and death, accelerating myocardial regeneration, and reducing fibrosis (Zhu K. et al., 2023).

While Allijn et al. demonstrated improved cardiac function preservation through liposomal berberine encapsulation, their study did not investigate the long-term stability and potential off-target effects beyond 28 days post-myocardial infarction, limiting comprehensive safety assessments (Allijn et al., 2017). Notably, Zhu et al. addressed nanoparticle delivery but did not quantitatively validate the *In vivo* drug release kinetics within the infarcted myocardium, leaving uncertainties regarding sustained therapeutic efficacy and dose optimization under physiological conditions (Zhu K. et al., 2023).

### 5.7 Tanshinone IIA

Tanshinone IIA, a lipophilic diterpenoid quinone (chemical formula C_19_H_18_O_3_, molecular weight 294.35 Da), is unambiguously identified as a secondary metabolite derived from the dried roots and rhizomes of *S. miltiorrhiza* Bunge (Lamiaceae). This compound exerts core cardiovascular protective effects, including anti-atherosclerotic activity, myocardial ischemic protection, and platelet aggregation inhibition, through mechanisms involving suppression of the NF-κB inflammatory pathway and modulation of lipid metabolism (Gao et al., 2012). Tanshinone IIA exerts its cardioprotective effects in MIRI through various mechanisms, including scavenging ROS, inhibiting the NF-κB signaling pathway to reduce inflammation, regulating apoptosis-related proteins to prevent myocardial cell death, protecting mitochondrial function to mitigate oxidative stress, and preventing myocardial fibrosis to prevent additional damage to cardiac function (Zhu P.-C. et al., 2023). Through these multi-pathway, multi-target actions, tanshinone IIA’s strong cardioprotective ability efficiently reduced cardiac damage induced by MIRI.

However, the poor solubility and low bioavailability of tanshinone IIA limit its clinical application (Huang et al., 2022). To address these challenges, researchers have employed nanocarrier technologies to enhance the pharmacological properties of carriers. One study developed a lipid-polymer nanocarrier system loaded with tanshinone IIA, modified with triphenylphosphine and D-α-tocopherol polyethylene glycol succinate, for targeted mitochondrial therapy in myocardial infarction. This system demonstrated significantly improved compatibility and therapeutic efficacy compared with free drugs and other similar nanocarriers in both *in vitro* and *in vivo* experiments. Pharmacokinetic and biodistribution studies confirmed the superior therapeutic effects of this nanocarrier system (Zhang et al., 2018).

Focusing on their effects on left ventricular remodeling after myocardial infarction, researchers in another study created monomethoxy polyethylene glycol-polylactic acid-D-α-tocopherol polyelsky nanoparticles containing tanshinone IIA. Within 4 weeks following myocardial infarction, tanshinone IIA nanoparticle treatment significantly recovered cardiac function, decreased infarct size, and effectively averted left ventricular dilatation in a mouse model. By lowering inhibitor of nuclear factor kappa B phosphorylation and the NF-κB signaling system, this drug clearly lowered cardiac inflammation, death, and fibrosis. Following myocardial infarction, tanshinone IIA nanoparticles lowered inhibitor of nuclear factor kappa B phosphorylation and NF-κB activity, hence improving heart remodeling (Mao et al., 2018). While the study demonstrates promising cardioprotective efficacy, its acute-phase dosing protocol (5-day post-MI administration) may be insufficient to mitigate persistent inflammatory and fibrotic cascades underlying chronic cardiac remodeling (Mao et al., 2018).

## 6 Discussion

Significant progress has been made in the treatment of MIRI using PDSM nanoparticles (Li et al., 2020; Li et al., 2022). The combination of modern nanotechnology with botanical drugs in this new strategy provides synergistic benefits for several targets and pathways (Chen et al., 2024). These nanoparticles overcome the main limitations of botanical drug treatments, such as restricted absorption and targeting (Liu C.-J. et al., 2021), thus improving therapeutic efficacy. Although initial results showed promise, considerable obstacles still exist in the therapeutic use of PDSM nanoparticles.

A key advantage of PDSM nanoparticles is their ability to exert synergistic effects across several targets and processes. Although many botanical drugs function through various pathways, PDSMs combined with nanotechnology provide significant benefits by simultaneously targeting many avenues. This technique enables the complete regulation of pathogenic processes, such as antioxidation, anti-inflammation, apoptosis suppression, and mitochondrial function modification. In MIRI models, quercetin-loaded nanoparticles demonstrated strong antioxidant effects, reducing oxidative stress, myocardial apoptosis, and inflammation while promoting angiogenesis and cardiac repair via the JAK2/STAT3 signaling pathway (Liu C.-J. et al., 2021). *Panax notoginseng* saponin-loaded nanoparticles decrease inflammation by blocking TNF-α and IL-6, activating the AKT and MAPK pathways, and reducing oxidative stress. As a result, post-reperfusion heart function has greatly improved (Li H. et al., 2022). Ginsenoside nanoparticles provide further cardioprotection by inhibiting apoptosis via the FoxO3a signaling pathway, as well as antioxidative and anti-inflammatory effects. This multifaceted approach makes PDSM nanoparticles a more effective therapeutic option for MIRI than traditional medicines.

The incorporation of nanotechnology has markedly enhanced the efficacy of the targeted delivery of PDSMs for MIRI treatment. Nanoparticles can precisely deliver drugs to damaged cardiac tissues while minimizing adverse effects in healthy areas by integrating passive and active targeting mechanisms. For instance, when vascular permeability increases during MIRI, nanoparticles using the EPR effect effectively aggregate in the injured regions (Lan et al., 2022). Drug localization can also be enhanced by active targeting strategies using nanoparticles covered with antibodies or peptides. Using external magnetic fields, magnetic targeting drives nanoparticles to the location of the injury, thereby increasing their therapeutic effects (Wei et al., 2024). By lowering the medication levels in non-targeted organs and reducing oxidative stress, mitochondria-targeted nanoparticles protect the mitochondria. This concentrated approach lowers adverse effects and increases medical efficacy (Gao et al., 2022).

Because they target many MIRI pathogen routes, multifunctional nanocarriers have synergistic therapeutic potential. By delivering antioxidants and anti-inflammatory medicines, these nanocarriers can effectively address several aspects of disease (Yang et al., 2023). For instance, while reducing inflammation and cardiomyocyte injury, nanoparticle solutions combining the EPR effect with tailored modifications can enhance drug delivery efficacy (Sun et al., 2012). Particularly pH- or ROS-responsive systems, liposomes, and polymeric nanoparticles provide precise control over drug release, thereby enhancing therapeutic effectiveness and reducing adverse effects (Li et al., 2020). Using these multifunctional nanocarriers in MIRI treatment showed their ability to cooperate across various systems, including anti-inflammation, anti-oxidation, and anti-apoptosis systems, thus producing considerably superior myocardial damage interventions.

Although PDSMs nanoparticles have shown potential therapeutic advantages in animal studies, various obstacles limit their clinical use. The long-term safety of nanocarriers requires further confirmation through clinical research, mostly in terms of their metabolism, excretion, and possible effects on other organs (Piscatelli et al., 2021). The complexity and variety of botanical drug constituents can impact the stability and efficacy of nanoparticles in clinical applications (Wei et al., 2022). Moreover, improving the pharmaceutical distribution and release techniques is a major challenge. Addressing the critical challenges in the clinical translation of botanical nanomedicines requires strategic advancements across material innovation, pharmacological optimization, and translational validation. In biodegradable carrier development, biocompatible polymers such as PLGA and natural polysaccharides are modified through surface engineering techniques (e.g., PEGylation) to reduce immunogenicity (Chatterjee and Chanda, 2022), with parallel long-term toxicological evaluations to assess organ-specific biodistribution profiles. Exploration of plant-derived natural vesicles, including exosomes and polysaccharide-based carriers, demonstrates potential for minimizing xenobiotic toxicity compared to synthetic alternatives (Basyoni et al., 2025; Chai et al., 2025). Following HPLC-MS-guided phytochemical screening and metabolomics-optimized drug loading (Plumb et al., 2023), nanocarriers are functionalized with immunomodulatory agents (e.g., siRNA targeting inflammatory mediators) to activate therapeutic pathways such as cGAS-STING signaling (Nagarajan et al., 2023). Translational validation employs 3D human organoid models and large-animal disease prototypes to quantify targeting specificity and biosafety parameters, integrated with standardized good manufacturing practice (GMP) protocols. Advanced pathology-responsive release systems, including oxidative stress-activated formulations, enable localized drug enrichment while maintaining systemic exposure below toxicity thresholds (Zheng et al., 2022).

Future studies should focus on integrating biomimetic nanotechnology with PDSMs to enhance biocompatibility and targeted efficacy. Encapsulating nanoparticles with myocardial or immune cell membranes may facilitate immune clearance and improve drug retention in the heart tissue (Liu et al., 2020; Zhou et al., 2023). This biomimetic approach improves the precise distribution of PDSM nanoparticles, enhancing their therapeutic efficacy in MIRI treatment. Moreover, the development of multifunctional hybrid nanoparticles has enhanced their potential for multitarget treatment. Future research should investigate how nanoparticles can concurrently carry genes, proteins, and other therapeutic compounds to address the complex disease processes of MIRI (Wang et al., 2021). For example, nanoparticle platforms integrating miRNA delivery methods have shown considerable promise in supporting myocardial regeneration and repair, indicating a possible breakthrough in the future treatment of cardiovascular disorders (Tan et al., 2021).

## 7 Conclusion

The clinical prospects of PDSM nanoparticles in the treatment of MIRI are highly promising. Nanotechnology not only boosts the bioavailability of bioactive phytochemicals but also enhances therapeutic outcomes through multi-target synergistic effects. However, successful translation to clinical practice requires further human trials to confirm long-term safety and efficacy. With advances in nanotechnology, more PDSM nanoparticles are expected to enter clinical trials, offering diverse and precise treatment options for MIRI.

## Abbreviations

MIRI, Myocardial ischemia-reperfusion injury; PDSMs, Plant-derived secondary metabolites; EPR, Enhanced Permeability and Retention; ROS, Reactive oxygen species; PEG, Polyethylene glycol; RAGE, Receptor of Advanced Glycation Endproducts; TPP, Triphenylphosphonium; PLGA, Polylactic acid/glycolic acid; NGR1, Notoginsenoside R1.

## References

[B1] Abdulredha A. Abosaooda M. Al-Amran F. Hadi N. R. (2021). Berberine protests the heart from ischemic reperfusion injury via interference with oxidative and inflammatory pathways. Med. Arch. 75, 174–179. 10.5455/medarh.2021.75.174-179 34483445 PMC8385727

[B2] Alizadeh S. R. Ebrahimzadeh M. A. (2022). Quercetin derivatives: drug design, development, and biological activities, a review. Eur. J. Med. Chem. 229, 114068. 10.1016/j.ejmech.2021.114068 34971873

[B3] Allijn I. E. Czarny B. M. S. Wang X. Chong S. Y. Weiler M. da Silva A. E. (2017). Liposome encapsulated berberine treatment attenuates cardiac dysfunction after myocardial infarction. J. Control Release 247, 127–133. 10.1016/j.jconrel.2016.12.042 28065862

[B4] Bae S. Park M. Kang C. Dilmen S. Kang T. H. Kang D. G. (2016). Hydrogen peroxide-responsive nanoparticle reduces myocardial ischemia/reperfusion injury. J. Am. Heart Assoc. 5, e003697. 10.1161/JAHA.116.003697 27930351 PMC5210353

[B5] Basyoni A. E. Atta A. Salem M. M. Mohamed T. M. (2025). Harnessing exosomes for targeted drug delivery systems to combat brain cancer. Cancer Cell. Int. 25, 150. 10.1186/s12935-025-03731-z 40234973 PMC12001718

[B6] Boarescu P.-M. Boarescu I. Bocşan I. C. Gheban D. Bulboacă A. E. Nicula C. (2019a). Antioxidant and anti-inflammatory effects of curcumin nanoparticles on drug-induced acute myocardial infarction in diabetic rats. Antioxidants (Basel) 8, 504. 10.3390/antiox8100504 31652638 PMC6826579

[B7] Boarescu P.-M. Boarescu I. Bocşan I. C. Pop R. M. Gheban D. Bulboacă A. E. (2019b). Curcumin nanoparticles protect against isoproterenol induced myocardial infarction by alleviating myocardial tissue oxidative stress, electrocardiogram, and biological changes. Molecules 24, 2802. 10.3390/molecules24152802 31374848 PMC6696485

[B8] Chai M. Gao B. Wang S. Zhang L. Pei X. Yue B. (2025). Leveraging plant-derived nanovesicles for advanced nucleic acid-based gene therapy. Theranostics 15, 324–339. 10.7150/thno.104507 39744221 PMC11667239

[B9] Chang X. Zhang Q. Huang Y. Liu J. Wang Y. Guan X. (2024). Quercetin inhibits necroptosis in cardiomyocytes after ischemia-reperfusion via DNA-PKcs-SIRT5-orchestrated mitochondrial quality control. Phytother. Res. 38, 2496–2517. 10.1002/ptr.8177 38447978

[B10] Chatterjee M. Chanda N. (2022). Formulation of PLGA nano-carriers: specialized modification for cancer therapeutic applications. Mat. Adv. 3, 837–858. 10.1039/D1MA00600B

[B11] Chen C. Liu W. Gu X. Zhang L. Mao X. Chen Z. (2024). Baicalin-loaded Polydopamine modified ZIF-8 NPs inhibits myocardial ischemia/reperfusion injury in rats. J. Biomater. Sci. Polym. Ed. 35, 1863–1878. 10.1080/09205063.2024.2358640 38830010

[B12] Chen J.-T. (2020). Advances in ginsenosides. Biomolecules 10, 681. 10.3390/biom10050681 32354079 PMC7277500

[B13] Cui J. Wang X. Dong L. Wang Q. (2024a). Curcumin reduces myocardial ischemia-reperfusion injury, by increasing endogenous H2S levels and further modulating m6A. Mol. Biol. Rep. 51, 558. 10.1007/s11033-024-09478-6 38643323

[B14] Cui J.-K. Fan M. Wang Q. (2024b). Curcumin reduces hypoxia/reperfusion injury of cardiomyocytes byStimulating vascular endothelial cells to secrete FGF2. Comb. Chem. High. Throughput Screen 27, 2101–2109. 10.2174/0113862073239166231103102648 37957857

[B15] Ding Y. Li W. Peng S. Zhou G. Chen S. Wei Y. (2023). Puerarin protects against myocardial ischemia/reperfusion injury by inhibiting ferroptosis. Biol. Pharm. Bull. 46, 524–532. 10.1248/bpb.b22-00174 36696989

[B16] Donato M. Evelson P. Gelpi R. J. (2017). Protecting the heart from ischemia/reperfusion injury: an update on remote ischemic preconditioning and postconditioning. Curr. Opin. Cardiol. 32, 784–790. 10.1097/HCO.0000000000000447 28902715

[B17] Dong L. Shen Z. Chi H. Wang Y. Shi Z. Fang H. (2023). Research progress of Chinese medicine in the treatment of myocardial ischemia-reperfusion injury. Am. J. Chin. Med. 51, 1–17. 10.1142/S0192415X23500015 36437553

[B18] Dong L. Zhao Y. Luo J. Li X. Wang S. Li M. (2024). Carbon dots derived from curcumae radix and their heartprotective effect. IJN 19, 3315–3332. 10.2147/IJN.S444125 38617797 PMC11012788

[B19] Dong Z. Guo J. Xing X. Zhang X. Du Y. Lu Q. (2017). RGD modified and PEGylated lipid nanoparticles loaded with puerarin: formulation, characterization and protective effects on acute myocardial ischemia model. Biomed. Pharmacother. 89, 297–304. 10.1016/j.biopha.2017.02.029 28236703

[B20] Du L. Lu H. Xiao Y. Guo Z. Li Y. (2024). Protective effect and pharmacokinetics of dihydromyricetin nanoparticles on oxidative damage of myocardium. PLoS One 19, e0301036. 10.1371/journal.pone.0301036 38625956 PMC11020404

[B21] Feng X. Sureda A. Jafari S. Memariani Z. Tewari D. Annunziata G. (2019). Berberine in cardiovascular and metabolic diseases: from mechanisms to therapeutics. Theranostics 9, 1923–1951. 10.7150/thno.30787 31037148 PMC6485276

[B22] Gao F. Zhao Y. Zhang B. Xiao C. Sun Z. Gao Y. (2022). Mitochondrial targeted astaxanthin liposomes for myocardial ischemia-reperfusion injury based on oxidative stress. J. Biomater. Appl. 37, 303–314. 10.1177/08853282221087102 35403475

[B23] Gao S. Liu Z. Li H. Little P. J. Liu P. Xu S. (2012). Cardiovascular actions and therapeutic potential of tanshinone IIA. Atherosclerosis 220, 3–10. 10.1016/j.atherosclerosis.2011.06.041 21774934

[B24] Gao Y. Li Z. Sun M. Li H. Guo C. Cui J. (2010). Preparation, characterization, pharmacokinetics, and tissue distribution of curcumin nanosuspension with TPGS as stabilizer. Drug Dev. Ind. Pharm. 36, 1225–1234. 10.3109/03639041003695139 20545506

[B25] Han D. Wang F. Jiang Q. Qiao Z. Zhuang Y. An Q. (2024). Enhancing cardioprotection through neutrophil-mediated delivery of 18β-glycyrrhetinic acid in myocardial ischemia/reperfusion injury. Adv. Sci. (Weinh) 11, e2406124. 10.1002/advs.202406124 39264272 PMC11558124

[B26] Han Y. Wang H. Wang Y. Dong P. Jia J. Yang S. (2021). Puerarin protects cardiomyocytes from ischemia-reperfusion injury by upregulating LncRNA ANRIL and inhibiting autophagy. Cell. Tissue Res. 385, 739–751. 10.1007/s00441-021-03463-2 33963896

[B27] Hu F. Hu T. Qiao Y. Huang H. Zhang Z. Huang W. (2024). Berberine inhibits excessive autophagy and protects myocardium against ischemia/reperfusion injury via the RhoE/AMPK pathway. Int. J. Mol. Med. 53, 49. 10.3892/ijmm.2024.5373 38577949 PMC10999226

[B28] Huang Q. Yao Y. Wang Y. Li J. Chen J. Wu M. (2023). Ginsenoside Rb2 inhibits p300-mediated SF3A2 acetylation at lysine 10 to promote Fscn1 alternative splicing against myocardial ischemic/reperfusion injury. J. Adv. Res. S2090-1232 (23), 365–379. 10.1016/j.jare.2023.12.012 PMC11518965 38101749

[B29] Huang X. Deng H. Shen Q.-K. Quan Z.-S. (2022). Tanshinone IIA: pharmacology, total synthesis, and progress in structure-modifications. Curr. Med. Chem. 29, 1959–1989. 10.2174/0929867328666211108110025 34749607

[B30] Jia X. Shao W. Tian S. (2022). Berberine alleviates myocardial ischemia-reperfusion injury by inhibiting inflammatory response and oxidative stress: the key function of miR-26b-5p-mediated PTGS2/MAPK signal transduction. Pharm. Biol. 60, 652–663. 10.1080/13880209.2022.2048029 35311466 PMC8967400

[B31] Jing J. Fang S. Li Y. Liu W. Wang C. Lan Y. (2024). An enhanced cardio-protective effect of nanoparticles loaded with active components from Polygonum orientale L. against isoproterenol-induced myocardial ischemia in rats. Int. J. Pharm. 655, 124047. 10.1016/j.ijpharm.2024.124047 38531434

[B32] Kim H. Lee J. H. Kim J. E. Kim Y. S. Ryu C. H. Lee H. J. (2018). Micro-/nano-sized delivery systems of ginsenosides for improved systemic bioavailability. J. Ginseng Res. 42, 361–369. 10.1016/j.jgr.2017.12.003 29983618 PMC6026383

[B33] Lan M. Hou M. Yan J. Deng Q. Zhao Z. Lv S. (2022). Cardiomyocyte-targeted anti-inflammatory nanotherapeutics against myocardial ischemia reperfusion (IR) injury. Nano Res. 15, 9125–9134. 10.1007/s12274-022-4553-6 35915748 PMC9328183

[B34] Lei W. Yan Y. Ma Y. Jiang M. Zhang B. Zhang H. (2022). Notoginsenoside R1 regulates ischemic myocardial lipid metabolism by activating the AKT/mTOR signaling pathway. Front. Pharmacol. 13, 905092. 10.3389/fphar.2022.905092 35814216 PMC9257227

[B35] Li F. Li D. Tang S. Liu J. Yan J. Chen H. (2021a). Quercetin protects H9c2 cardiomyocytes against oxygen-glucose deprivation/reoxygenation-induced oxidative stress and mitochondrial apoptosis by regulating the ERK1/2/DRP1 signaling pathway. Evid. Based Complement. Altern. Med. 2021, 7522175. 10.1155/2021/7522175 PMC8390138 34457029

[B36] Li H. Li M. Fu J. Ao H. Wang W. Wang X. (2021b). Enhancement of oral bioavailability of quercetin by metabolic inhibitory nanosuspensions compared to conventional nanosuspensions. Drug Deliv. 28, 1226–1236. 10.1080/10717544.2021.1927244 34142631 PMC8218931

[B37] Li H. Zhu J. Xu Y.-W. Mou F.-F. Shan X.-L. Wang Q.-L. (2022a). Notoginsenoside R1-loaded mesoporous silica nanoparticles targeting the site of injury through inflammatory cells improves heart repair after myocardial infarction. Redox Biol. 54, 102384. 10.1016/j.redox.2022.102384 35777198 PMC9287735

[B38] Li L. Wang Y. Guo R. Li S. Ni J. Gao S. (2020). Ginsenoside Rg3-loaded, reactive oxygen species-responsive polymeric nanoparticles for alleviating myocardial ischemia-reperfusion injury. J. Control Release 317, 259–272. 10.1016/j.jconrel.2019.11.032 31783047 PMC7384207

[B39] Li M. Wu J. Yang T. Zhao Y. Ren P. Chang L. (2024). Engineered biomimetic nanoparticles-mediated targeting delivery of allicin against myocardial ischemia-reperfusion injury by inhibiting ferroptosis. Int. J. Nanomedicine 19, 11275–11292. 10.2147/IJN.S478276 39524923 PMC11550785

[B40] Li X. Wang G. Sun J. Hao H. Xiong Y. Yan B. (2007). Pharmacokinetic and absolute bioavailability study of total panax notoginsenoside, a typical multiple constituent traditional Chinese medicine (TCM) in rats. Biol. Pharm. Bull. 30, 847–851. 10.1248/bpb.30.847 17473424

[B41] Li Y. Hao H. Yu H. Yu L. Ma H. Zhang H. (2022b). Ginsenoside Rg2 ameliorates myocardial ischemia/reperfusion injury by regulating TAK1 to inhibit necroptosis. Front. Cardiovasc Med. 9, 824657. 10.3389/fcvm.2022.824657 35391841 PMC8981204

[B42] Liu C. Zhang X. Yang H. Zhao M. Liu Y. Zhao R. (2024). PEG-modified nano liposomes co-deliver Apigenin and RAGE-siRNA to protect myocardial ischemia injury. Int. J. Pharm. 649, 123673. 10.1016/j.ijpharm.2023.123673 38056796

[B43] Liu C.-J. Yao L. Hu Y.-M. Zhao B.-T. (2021a). Effect of quercetin-loaded mesoporous silica nanoparticles on myocardial ischemia-reperfusion injury in rats and its mechanism. IJN 16, 741–752. 10.2147/IJN.S277377 33564233 PMC7866914

[B44] Liu Y. Li L. Wang Z. Zhang J. Zhou Z. (2023). Myocardial ischemia-reperfusion injury; Molecular mechanisms and prevention. Microvasc. Res. 149, 104565. 10.1016/j.mvr.2023.104565 37307911

[B45] Liu Y. Song Y. Li S. Mo L. (2021b). Cardioprotective effect of quercetin against ischemia/reperfusion injury is mediated through NO system and mitochondrial K-ATP channels. Cell. J. 23, 184–190. 10.22074/cellj.2021.7183 34096219 PMC8181321

[B46] Liu Y. Xie X. Chen H. Hou X. He Y. Shen J. (2020). Advances in next-generation lipid-polymer hybrid nanocarriers with emphasis on polymer-modified functional liposomes and cell-based-biomimetic nanocarriers for active ingredients and fractions from Chinese medicine delivery. Nanomedicine 29, 102237. 10.1016/j.nano.2020.102237 32534047

[B47] Long T. Pan W. Li F. Sheikh S. A. Xie Q. Zhang C. (2023). Berberine up-regulates miR-340-5p to protect myocardial ischaemia/reperfusion from HMGB1-mediated inflammatory injury. Esc. Heart Fail 10, 931–942. 10.1002/ehf2.14235 36453191 PMC10053273

[B48] Lozano O. Lázaro-Alfaro A. Silva-Platas C. Oropeza-Almazán Y. Torres-Quintanilla A. Bernal-Ramírez J. (2019). Nanoencapsulated quercetin improves cardioprotection during hypoxia-reoxygenation injury through preservation of mitochondrial function. Oxid. Med. Cell. Longev. 2019, 7683051. 10.1155/2019/7683051 31341535 PMC6612997

[B49] Mao S. Wang L. Chen P. Lan Y. Guo R. Zhang M. (2018). Nanoparticle-mediated delivery of Tanshinone IIA reduces adverse cardiac remodeling following myocardial infarctions in a mice model: role of NF-κB pathway. Artif. Cells Nanomed Biotechnol. 46, S707–S716. 10.1080/21691401.2018.1508028 30284484

[B50] Mi X. Hu M. Dong M. Yang Z. Zhan X. Chang X. (2021). Folic acid decorated zeolitic imidazolate framework (ZIF-8) loaded with baicalin as a nano-drug delivery system for breast cancer therapy. Int. J. Nanomedicine 16, 8337–8352. 10.2147/IJN.S340764 34992370 PMC8714011

[B51] Nabofa W. E. E. Alashe O. O. Oyeyemi O. T. Attah A. F. Oyagbemi A. A. Omobowale T. O. (2018). Cardioprotective effects of curcumin-nisin based poly lactic acid nanoparticle on myocardial infarction in Guinea pigs. Sci. Rep. 8, 16649. 10.1038/s41598-018-35145-5 30413767 PMC6226538

[B52] Nagarajan A. G. Neeley N. Doroodian P. Olazabel A. Kupfer G. (2023). Genome wide siRNA screen identifies CGAS-sting pathway as a pharmacological target that promotes survival of hematopoietic stem cells deficient in fanconi genes. Blood 142, 1083. 10.1182/blood-2023-187180

[B53] Pan W. Xue B. Yang C. Miao L. Zhou L. Chen Q. (2018). Biopharmaceutical characters and bioavailability improving strategies of ginsenosides. Fitoterapia 129, 272–282. 10.1016/j.fitote.2018.06.001 29883635

[B54] Pei H. Wu Y. Wei Y. Yang Y. Teng S. Zhang H. (2014). Remote ischemic preconditioning reduces perioperative cardiac and renal events in patients undergoing elective coronary intervention: a meta-analysis of 11 randomized trials. PLoS One 9, e115500. 10.1371/journal.pone.0115500 25551671 PMC4281209

[B55] Piscatelli J. A. Ban J. Lucas A. T. Zamboni W. C. (2021). Complex factors and challenges that affect the pharmacology, safety and efficacy of nanocarrier drug delivery systems. Pharmaceutics 13, 114. 10.3390/pharmaceutics13010114 33477395 PMC7830329

[B56] Plumb R. S. Gethings L. A. Rainville P. D. Isaac G. Trengove R. King A. M. (2023). Advances in high throughput LC/MS based metabolomics: a review. TrAC Trends Anal. Chem. 160, 116954. 10.1016/j.trac.2023.116954

[B57] Qin G.-W. Lu P. Peng L. Jiang W. (2021). Ginsenoside Rb1 inhibits cardiomyocyte autophagy via PI3K/Akt/mTOR signaling pathway and reduces myocardial ischemia/reperfusion injury. Am. J. Chin. Med. 49, 1913–1927. 10.1142/S0192415X21500907 34775933

[B58] Qiu J. Cai G. Liu X. Ma D. (2017). αvβ3 integrin receptor specific peptide modified, salvianolic acid B and panax notoginsenoside loaded nanomedicine for the combination therapy of acute myocardial ischemia. Biomed. Pharmacother. 96, 1418–1426. 10.1016/j.biopha.2017.10.086 29079344

[B59] Safiri S. Karamzad N. Singh K. Carson-Chahhoud K. Adams C. Nejadghaderi S. A. (2022). Burden of ischemic heart disease and its attributable risk factors in 204 countries and territories, 1990-2019. Eur. J. Prev. Cardiol. 29, 420–431. 10.1093/eurjpc/zwab213 34922374

[B60] Sarhene M. Ni J. Y. Duncan E. S. Liu Z. Li S. Zhang J. (2021). Ginsenosides for cardiovascular diseases; update on pre-clinical and clinical evidence, pharmacological effects and the mechanisms of action. Pharmacol. Res. 166, 105481. 10.1016/j.phrs.2021.105481 33549726

[B61] Schäfer A. König T. Bauersachs J. Akin M. (2022). Novel therapeutic strategies to reduce reperfusion injury after acute myocardial infarction. Curr. Probl. Cardiol. 47, 101398. 10.1016/j.cpcardiol.2022.101398 36108813

[B62] Song D. Hao J. Fan D. (2020). Biological properties and clinical applications of berberine. Front. Med. 14, 564–582. 10.1007/s11684-019-0724-6 32335802

[B63] Sun G. Lin X. Hong Y. Feng Y. Ruan K. Xu D. (2012). PEGylation for drug delivery to ischemic myocardium: pharmacokinetics and cardiac distribution of poly(ethylene glycol)s in mice with normal and ischemic myocardium. Eur. J. Pharm. Sci. 46, 545–552. 10.1016/j.ejps.2012.04.010 22525436

[B64] Tan H. Song Y. Chen J. Zhang N. Wang Q. Li Q. (2021). Platelet-like fusogenic liposome-mediated targeting delivery of miR-21 improves myocardial remodeling by reprogramming macrophages post myocardial ischemia-reperfusion injury. Adv. Sci. (Weinh) 8, e2100787. 10.1002/advs.202100787 34137511 PMC8336489

[B65] Tong Q. Zhu P.-C. Zhuang Z. Deng L.-H. Wang Z.-H. Zeng H. (2019). Notoginsenoside R1 for organs ischemia/reperfusion injury: a preclinical systematic review. Front. Pharmacol. 10, 1204. 10.3389/fphar.2019.01204 31680976 PMC6811647

[B66] Upadhaya S. Madala S. Baniya R. Subedi S. K. Saginala K. Bachuwa G. (2017). Impact of cyclosporine A use in the prevention of reperfusion injury in acute myocardial infarction: a meta-analysis. Cardiol. J. 24, 43–50. 10.5603/CJ.a2016.0091 27734457

[B67] Voronkov N. S. Popov S. V. Naryzhnaya N. V. Prasad N. R. Petrov I. M. Kolpakov V. V. (2024). Effect of cold adaptation on the state of cardiovascular system and cardiac tolerance to ischemia/reperfusion injury. Iran. Biomed. J. 28, 59–70. 10.61186/ibj.3872 38770843 PMC11186613

[B68] Wang D. Bu T. Li Y. He Y. Yang F. Zou L. (2022). Pharmacological activity, pharmacokinetics, and clinical research progress of puerarin. Antioxidants (Basel) 11, 2121. 10.3390/antiox11112121 36358493 PMC9686758

[B69] Wang J. Liu Y. Liu Y. Huang H. Roy S. Song Z. (2023a). Recent advances in nanomedicines for imaging and therapy of myocardial ischemia-reperfusion injury. J. Control Release 353, 563–590. 10.1016/j.jconrel.2022.11.057 36496052

[B70] Wang K. Feng X. Chai L. Cao S. Qiu F. (2017). The metabolism of berberine and its contribution to the pharmacological effects. Drug Metab. Rev. 49, 139–157. 10.1080/03602532.2017.1306544 28290706

[B71] Wang Q. Song Y. Chen J. Li Q. Gao J. Tan H. (2021). Direct *in vivo* reprogramming with non-viral sequential targeting nanoparticles promotes cardiac regeneration. Biomaterials 276, 121028. 10.1016/j.biomaterials.2021.121028 34293701

[B72] Wang Y. Li F. Wei S. Li W. Wu J. Li S. (2024a). Puerarin-loaded liposomes Co-modified by ischemic myocardium-targeting peptide and triphenylphosphonium cations ameliorate myocardial ischemia-reperfusion injury. Int. J. Nanomedicine 19, 7997–8014. 10.2147/IJN.S468394 39130683 PMC11317047

[B73] Wang Y. Li S. Li W. Wu J. Hu X. Tang T. (2024b). Cardiac-targeted and ROS-responsive liposomes containing puerarin for attenuating myocardial ischemia-reperfusion injury. Nanomedicine (Lond) 19, 2335–2355. 10.1080/17435889.2024.2402678 39316570 PMC11492708

[B74] Wang Y. Wang Q. Wang X. Yao P. Dai Q. Qi X. (2023b). Docetaxel-loaded pH/ROS dual-responsive nanoparticles with self-supplied ROS for inhibiting metastasis and enhancing immunotherapy of breast cancer. J. Nanobiotechnology 21, 286. 10.1186/s12951-023-02013-y 37608285 PMC10464340

[B75] Wang Y. Wang Y. Wang X. Hu P. (2018). Tilianin-loaded reactive oxygen species-scavenging nano-micelles protect H9c2 cardiomyocyte against hypoxia/reoxygenation-induced injury. J. Cardiovasc Pharmacol. 72, 32–39. 10.1097/FJC.0000000000000587 29688912

[B76] Wang Y. Zheng J. Xiao X. Feng C. Li Y. Su H. (2024c). Ginsenoside Rd attenuates myocardial ischemia/reperfusion injury by inhibiting inflammation and apoptosis through PI3K/akt signaling pathway. Am. J. Chin. Med. 52, 433–451. 10.1142/S0192415X24500186 38577825

[B77] Wang Z.-K. Chen R.-R. Li J.-H. Chen J.-Y. Li W. Niu X.-L. (2020). Puerarin protects against myocardial ischemia/reperfusion injury by inhibiting inflammation and the NLRP3 inflammasome: the role of the SIRT1/NF-κB pathway. Int. Immunopharmacol. 89, 107086. 10.1016/j.intimp.2020.107086 33068868

[B78] Wei D. Yang H. Zhang Y. Zhang X. Wang J. Wu X. (2022). Nano-traditional Chinese medicine: a promising strategy and its recent advances. J. Mater Chem. B 10, 2973–2994. 10.1039/D2TB00225F 35380567

[B79] Wei Q. Xiao Y. Du L. Li Y. (2024). Advances in nanoparticles in the prevention and treatment of myocardial infarction. Molecules 29, 2415. 10.3390/molecules29112415 38893291 PMC11173599

[B80] Welt F. G. P. Batchelor W. Spears J. R. Penna C. Pagliaro P. Ibanez B. (2024). Reperfusion injury in patients with acute myocardial infarction: JACC scientific statement. J. Am. Coll. Cardiol. 83, 2196–2213. 10.1016/j.jacc.2024.02.056 38811097

[B81] Wu H.-J. Zhang K. Ma J.-J. Wang L. Zhuang Y. (2021). Mechanism of curcumin against myocardial ischaemia-reperfusion injury based on the P13K/Akt/mTOR signalling pathway. Eur. Rev. Med. Pharmacol. Sci. 25, 5490–5499. 10.26355/eurrev_202109_26658 34533799

[B82] Xie Y. Shen X. Xu F. Liang X. (2024). Research progress of nano-delivery systems for the active ingredients from traditional Chinese medicine. Phytochem. Anal. 10.1002/pca.3381 38830775

[B83] Xu H. Hu M. Liu M. An S. Guan K. Wang M. (2020). Nano-puerarin regulates tumor microenvironment and facilitates chemo- and immunotherapy in murine triple negative breast cancer model. Biomaterials 235, 119769. 10.1016/j.biomaterials.2020.119769 31986348 PMC7093100

[B84] Xu H. Zhang X. Shi Y. Yu K. Jiang Y. (2022). Notoginsenoside R1 relieves the myocardial infarction via activating the JAK2/STAT3 signaling pathway *in vivo* and *in vitro* . Bioengineered 13, 5653–5662. 10.1080/21655979.2022.2037366 35263202 PMC8974102

[B85] Xu H.-X. Pan W. Qian J.-F. Liu F. Dong H.-Q. Liu Q.-J. (2019). MicroRNA-21 contributes to the puerarin-induced cardioprotection via suppression of apoptosis and oxidative stress in a cell model of ischemia/reperfusion injury. Mol. Med. Rep. 20, 719–727. 10.3892/mmr.2019.10266 31115556

[B86] Xu S. Wu B. Zhong B. Lin L. Ding Y. Jin X. (2021). Naringenin alleviates myocardial ischemia/reperfusion injury by regulating the nuclear factor-erythroid factor 2-related factor 2 (Nrf2)/System xc-/glutathione peroxidase 4 (GPX4) axis to inhibit ferroptosis. Bioengineered 12, 10924–10934. 10.1080/21655979.2021.1995994 34699317 PMC8809912

[B87] Xu X. Diao Z. Zhao B. Xu H. Yan S. Chen H. (2024). Protective activities of silver nanoparticles containing *Panax japonicus* on apoptotic, inflammatory, and oxidative alterations in isoproterenol-induced cardiotoxicity. Open Chem. 22, 20240006. 10.1515/chem-2024-0006

[B88] Xue Y. Fu W. Liu Y. Yu P. Sun M. Li X. (2020). Ginsenoside Rb2 alleviates myocardial ischemia/reperfusion injury in rats through SIRT1 activation. J. Food Sci. 85, 4039–4049. 10.1111/1750-3841.15505 33073372

[B89] Xue Y. Fu W. Yu P. Li Y. Yu X. Xu H. (2023). Ginsenoside Rc alleviates myocardial ischemia-reperfusion injury by reducing mitochondrial oxidative stress and apoptosis: role of SIRT1 activation. J. Agric. Food Chem. 71, 1547–1561. 10.1021/acs.jafc.2c06926 36626267

[B90] Yalikong A. Li X.-Q. Zhou P.-H. Qi Z.-P. Li B. Cai S.-L. (2021). A triptolide loaded HER2-targeted nano-drug delivery system significantly suppressed the proliferation of HER2-positive and BRAF mutant colon cancer. Int. J. Nanomedicine 16, 2323–2335. 10.2147/IJN.S287732 33776436 PMC7989962

[B91] Yan L. Pan C.-S. Liu Y.-Y. Cui Y.-C. Hu B.-H. Chang X. (2021). The composite of 3, 4-dihydroxyl-phenyl lactic acid and notoginsenoside R1 attenuates myocardial ischemia and reperfusion injury through regulating mitochondrial respiratory chain. Front. Physiol. 12, 538962. 10.3389/fphys.2021.538962 34322032 PMC8311465

[B92] Yan S. Yue Y. Zeng L. Jiang C. Li W. Li H. (2019). Ligustrazine nanoparticles nano spray’s activation on Nrf2/ARE pathway in oxidative stress injury in rats with postoperative abdominal adhesion. Ann. Transl. Med. 7, 379. 10.21037/atm.2019.07.72 31555693 PMC6736816

[B93] Yang C. Yang S. Fang S. Li L. Jing J. Liu W. (2023). PLGA nanoparticles enhanced cardio-protection of scutellarin and paeoniflorin against isoproterenol-induced myocardial ischemia in rats. Int. J. Pharm. 648, 123567. 10.1016/j.ijpharm.2023.123567 37918495

[B94] Yang K.-T. Chao T.-H. Wang I.-C. Luo Y.-P. Ting P.-C. Lin J.-H. (2022). Berberine protects cardiac cells against ferroptosis. Tzu Chi Med. J. 34, 310–317. 10.4103/tcmj.tcmj_236_21 35912047 PMC9333108

[B95] Yao C. Shi X. Lin X. Shen L. Xu D. Feng Y. (2015). Increased cardiac distribution of mono-PEGylated Radix Ophiopogonis polysaccharide in both myocardial infarction and ischemia/reperfusion rats. Int. J. Nanomedicine 10, 409–418. 10.2147/IJN.S73462 25609953 PMC4298336

[B96] Ye J. Lyu T.-J. Li L.-Y. Liu Y. Zhang H. Wang X. (2023). Ginsenosi*de re* attenuates myocardial ischemia/reperfusion induced ferroptosis via miR-144-3p/SLC7A11. Phytomedicine 113, 154681. 10.1016/j.phymed.2023.154681 36893674

[B97] Yu W. Ding J. Chen J. Jiang Y. Zhao J. Liu J. (2024). Magnesium ion-doped mesoporous bioactive glasses loaded with gallic acid against myocardial ischemia/reperfusion injury by affecting the biological functions of multiple cells. Int. J. Nanomedicine 19, 347–366. 10.2147/IJN.S444751 38229705 PMC10790657

[B98] Zeng J.-J. Shi H.-Q. Ren F.-F. Zhao X.-S. Chen Q.-Y. Wang D.-J. (2023). Notoginsenoside R1 protects against myocardial ischemia/reperfusion injury in mice via suppressing TAK1-JNK/p38 signaling. Acta Pharmacol. Sin. 44, 1366–1379. 10.1038/s41401-023-01057-y 36721009 PMC10310839

[B99] Zhang J. Han X. Li X. Luo Y. Zhao H. Yang M. (2012). Core-shell hybrid liposomal vesicles loaded with panax notoginsenoside: preparation, characterization and protective effects on global cerebral ischemia/reperfusion injury and acute myocardial ischemia in rats. Int. J. Nanomedicine 7, 4299–4310. 10.2147/IJN.S32385 22915851 PMC3419509

[B100] Zhang J. Liu S. Jiang L. Hou J. Yang Z. (2022). Curcumin improves cardiopulmonary resuscitation outcomes by modulating mitochondrial metabolism and apoptosis in a rat model of cardiac arrest. Front. Cardiovasc Med. 9, 908755. 10.3389/fcvm.2022.908755 35665263 PMC9160380

[B101] Zhang S. Li J. Hu S. Wu F. Zhang X. (2018). Triphenylphosphonium and D-α-tocopheryl polyethylene glycol 1000 succinate-modified, tanshinone IIA-loaded lipid-polymeric nanocarriers for the targeted therapy of myocardial infarction. Int. J. Nanomedicine 13, 4045–4057. 10.2147/IJN.S165590 30022826 PMC6045899

[B102] Zhang S. Xia J. Zhu Y. Dong M. Wang J. (2024). Establishing Salvia miltiorrhiza-derived exosome-like nanoparticles and elucidating their role in angiogenesis. Molecules 29, 1599. 10.3390/molecules29071599 38611878 PMC11013048

[B103] Zhang Y.-M. Zhang Z.-Y. Wang R.-X. (2020). Protective mechanisms of quercetin against myocardial ischemia reperfusion injury. Front. Physiol. 11, 956. 10.3389/fphys.2020.00956 32848878 PMC7412593

[B104] Zheng Y. Wang Y. Xia M. Gao Y. Zhang L. Song Y. (2022). The combination of nanotechnology and traditional Chinese medicine (TCM) inspires the modernization of TCM: review on nanotechnology in TCM-based drug delivery systems. Drug Deliv. Transl. Res. 12, 1306–1325. 10.1007/s13346-021-01029-x 34260049

[B105] Zhou Y. Liang Q. Wu X. Duan S. Ge C. Ye H. (2023). siRNA delivery against myocardial ischemia reperfusion injury mediated by reversibly camouflaged biomimetic nanocomplexes. Adv. Mater 35, e2210691. 10.1002/adma.202210691 36913720

[B106] Zhou Y.-X. Zhang H. Peng C. (2014). Puerarin: a review of pharmacological effects. Phytother. Res. 28, 961–975. 10.1002/ptr.5083 24339367

[B107] Zhou Y.-X. Zhang H. Peng C. (2021). Effects of puerarin on the prevention and treatment of cardiovascular diseases. Front. Pharmacol. 12, 771793. 10.3389/fphar.2021.771793 34950032 PMC8689134

[B108] Zhu K. Yao Y. Wang K. Shao F. Zhu Z. Song Y. (2023a). Berberin sustained-release nanoparticles were enriched in infarcted rat myocardium and resolved inflammation. J. Nanobiotechnol 21, 33. 10.1186/s12951-023-01790-w PMC9883926 36709291

[B109] Zhu P.-C. Shen J. Qian R.-Y. Xu J. Liu C. Hu W.-M. (2023b). Effect of tanshinone IIA for myocardial ischemia/reperfusion injury in animal model: preclinical evidence and possible mechanisms. Front. Pharmacol. 14, 1165212. 10.3389/fphar.2023.1165212 37261285 PMC10228700

[B110] Zhu T. Wan Q. (2023). Pharmacological properties and mechanisms of Notoginsenoside R1 in ischemia-reperfusion injury. Chin. J. Traumatol. 26, 20–26. 10.1016/j.cjtee.2022.06.008 35922249 PMC9912185

[B111] Zhu Y. Yue M. Guo T. Li F. Li Z. Yang D. (2021). PEI-PEG-Coated mesoporous silica nanoparticles enhance the antitumor activity of tanshinone IIA and serve as a gene transfer vector. Evid. Based Complement. Altern. Med. 2021, 6756763. 10.1155/2021/6756763 PMC8592735 34790248

